# Dopamine Receptor Subtypes, Physiology and Pharmacology: New Ligands and Concepts in Schizophrenia

**DOI:** 10.3389/fphar.2020.01003

**Published:** 2020-07-14

**Authors:** Jean Claude Martel, Silvia Gatti McArthur

**Affiliations:** ^1^ Independent Researcher, Amos, QC, Canada; ^2^ McArthur and Associates, Basel, Switzerland

**Keywords:** schizophrenia, dopamine receptor, NMDA, antipsychotic, psychosis, D1, D2, D3

## Abstract

Dopamine receptors are widely distributed within the brain where they play critical modulator roles on motor functions, motivation and drive, as well as cognition. The identification of five genes coding for different dopamine receptor subtypes, pharmacologically grouped as D1- (D1 and D5) or D2-like (D2S, D2L, D3, and D4) has allowed the demonstration of differential receptor function in specific neurocircuits. Recent observation on dopamine receptor signaling point at dopamine—glutamate-NMDA neurobiology as the most relevant in schizophrenia and for the development of new therapies. Progress in the chemistry of D1- and D2-like receptor ligands (agonists, antagonists, and partial agonists) has provided more selective compounds possibly able to target the dopamine receptors homo and heterodimers and address different schizophrenia symptoms. Moreover, an extensive evaluation of the functional effect of these agents on dopamine receptor coupling and intracellular signaling highlights important differences that could also result in highly differentiated clinical pharmacology. The review summarizes the recent advances in the field, addressing the relevance of emerging new targets in schizophrenia in particular in relation to the dopamine – glutamate NMDA systems interactions.

## Introduction

The dopaminergic system undergoes a delayed maturation in the brain, suggesting important stabilizing and integrating functions on neural circuits (Grace, 2016; Ohira, 2020). Schizophrenia (SCZ) is associated with dopamine (DA) neurotransmission alterations during puberty and adult life causing deficits in motivation, cognition and sensory functions (Simpson and Kellendonk, 2017; Abi-Dargham, 2018; Grace and Gomes, 2019; Sonnenschein and Grace, 2020). DA release measures in SCZ clinical studies and in preclinical models have clearly documented a fronto-cortical DA hypoactivity and a striatal (mainly dorsal) DA hyperactivity, associated with the occurrence of different SCZ symptoms (Terrillion et al., 2017; McCutcheon et al., 2019; Rao et al., 2019; Li et al., 2020). A summary of the most recent experimental evidence linking SCZ to DA alterations can be found in **Table 1** (McCutcheon et al., 2020). Recent studies are however questioning the causal role of DA in SCZ in favor of a more “NMDA hypofunction hypothesis” of the disease. The limited SCZ genetic links to dopamine receptors (DR) and the main glutamatergic alterations observed in SCZ imaging studies are among the most compelling reasons for this debate (Coyle et al., 2010; McCutcheon et al., 2020) (see also supplementary material **Table 1** for genetic links). This clearly does not question the well documented therapeutic benefit of DR antagonists as antipsychotics, but challenges two decades of efforts to develop new and improved SCZ therapies. This review aims at providing a summary of the most recent advances in DR control in SCZ with focus on DR—glutamate NMDA interactions across the genetic, intracellula,r and synaptic aspects of the disease. (Rampino et al., 2018).

**Table 1:** Summary of most recent evidence of dopaminergic alterations in schizophrenia.

Method	Results	References
*Functional Imaging*	Impaired PFCx control. Dorsal striatum alterations	(McCutcheon et al., 2019)
*PET studies*	Increased DA synthesis - release. Reduction during symptoms remission/D2 occupancy of antipsychotics./Hypo DA in PFCx. Antipsychotic treatment response./Effect of stress and DA in the reward circuit/DA alterations and white matter reduction./Pre- and postsynaptic alterations./SCZ subtypes./High risk SCZ patients.	(Abi-Dargham, 2018; Mitelman et al., 2018; Tseng et al., 2018; Avram et al., 2019; D’Ambrosio et al., 2019; Kim et al., 2019; Rao et al., 2019; Sekiguchi et al., 2019; Weidenauer et al., 2020; Girgis et al., 2020; Brugger et al., 2020; Wulff et al., 2020; Frankle and Narendran, 2020)
*Post mortem*	DAT levels./Presynaptic dysregulation.	(Tseng et al., 2017; Purves-Tyson et al., 2017)
*Genetic/epigenetic*	DA sensitization in SCZ./NMDA DR epigenetic./Cumulative DA genetic and response inhibition./DR genetic variants and heterodimerization.	(Oishi et al., 2020; Enge et al., 2020; Faron-Gorecka et al., 2020; Jackson, 2020)
*Transcriptional*	SCZ risk genes control on D2 pathway expression.	(Torretta et al., 2020)
*Protein level*	Impact of DA on posttranslational control	(Kos et al., 2018)
*Developmental*	Netrin1/DCC on DA neuronal dev./MAM model.	(Grace and Gomes, 2019; Sonnenschein and Grace, 2020; Vosberg et al., 2020)
*Biomarker*	Anti-NMDA antibodies reduces D1 trafficking/Neuromelanin imaging.	(Grea et al., 2019; Wengler et al., 2020)
*Therapy*	Review on antipsychotics/Clinical effect of TAAR1 agonist.	(Koblan et al., 2020; Willner et al., 2020)
*Cognitive*	DA breakdown and working memory/D2 and cognition, area volumes -IQ./D2-like receptors and executive function.	(Bolton and Constantine-Paton, 2018; Veselinovic et al., 2018; Chang et al., 2020)
*Animal models*	Blonanserine in SCZ-like symptoms rodent models/DA alterations in rodents with NMDA hypofunction/model relevant for prodromal SCZ.	(Petty et al., 2019; Nakao et al., 2019; Takeuchi et al., 2019)
*Translational*	Extensive review from bentch to bed-side.	(Abi-Dargham, 2020)
*Pharmacology*	Lumateperone D1 and D2-like antipsychotic profile./Cariprazine new data.	(Vyas et al., 2020; Periclou et al., 2020)
*Morphology*	Rodent dorsal striatum synaptosome and Disc1	(Sialana et al., 2018)

## Section 1: Dopamine Receptors

### DA Neurophysiology

DA is a neurotransmitter produced in neuronal terminals by successive hydroxylation and decarboxylation of tyrosine and loaded into synaptic vesicles by the monoamine transporter 2 (VMAT2/SCL18A2). When glutamate is coreleased with DA, VGLUT2-mediated glutamate uptake causes vesicular acidification and increases DA packing (El Mestikawy et al., 2011). Released DA is targeted for reuptake by two solute carriers, DAT1/SLC6A3 and DAT/SLCA2, with a prevalence of the effect of DAT1. The degradation of DA is under the control of a methylation enzyme, COMT (highly expressed in prefrontal cortex) and presynaptic monoamine oxidases. The by-product of this oxidation, H_2_O_2_ is funneled into the mitochondrial transport chain to support further DA release (Chen and Jonas, 2020). DA release occurs in a rather diffuse manner and ultrastructural studies show DA neuron axonal arborization and intricate projections covering large areas. DA transmission is tightly controlled at presynaptic level, while only varicosity elements define the postsynaptic sites with a variety of inputs (cholinergic, glutamatergic) in close proximity. DA neurons are specialized to receive high volumes of afferent signals and transform this information into a modulatory tone through a large projection area. It is estimated that one DA neuron provides input to several thousand neurons in the striatum and vice-versa, any given individual striatal neuron is influenced by DA released from more than one hundred DA projections. The DA neuronal system is often described in terms of DA release (tonic or phasic) and several models have tried to explain how multiple functions can be effectively impacted by different temporal DA release patterns (Eshel et al., 2015; Berke, 2018; Lohani et al., 2019; Mohebi et al., 2019). DA neurons are intrinsic pacemakers, with a slow (2–4 Hz) rhythmic activity associated with a tonic feed-forward control on DA receptor activation. The ionic channels/voltage sensitive mechanisms controlling DA tonic firing activity can differ even in within each DA nucleus. DA neurons can also fire in rapid bursts in response to relevant (salient) stimuli. This transient increase in firing rate induces a temporally precise rise in DA concentrations that can be synchronized in within local circuits. The lack of canonical synaptic release sites and the low probability of release for DA containing vesicles allow a scaling of neurotransmitter release as a function firing frequencies (Lebowitz and Khoshbouei, 2020). DA neurons in normal conditions always contain a “reserve pool” of DA vesicles that are rather insensitive to stimulation and more than half of DA synaptic release sites are functionally silent when stimulated. The DA system is therefore also sensitive to a local presynaptic modulation from other neurotransmitters (like acetylcholine or endocannabinoids) (Xu et al., 2018). DAT exerts a main presynaptic master control on DA release as recently demonstrated (Condon et al., 2019; Walters et al., 2020). DA release is in fact directly modulated at the presynaptic terminals by a Rho-dependent internalization of DAT. This prolongs DA availability after burst stimulation, causing a prolonged postburst increase (>20 min) (Lohani et al., 2018). Differences in presynaptic Ca^2+^ channels and Ca^2+^ buffering further contribute to DA release synaptic heterogeneity (Chuhma et al., 2017). Large postexperience DA stimulation phases are important during learning procedures and in motivational drive, reward processes (Lak et al., 2020; Song and Lee, 2020). Most likely both D1 and D2 receptors subtypes are differentially engaged when in presence of DA burst firing at least in cortical and striatal regions (Hunger et al., 2020). Experimental evidence points at presynaptic alterations in DA nerve terminals in the striatal region and in prefrontal cortex in SCZ (Chuhma et al., 2017; McCutcheon et al., 2020; Weidenauer et al., 2020). Independent groups have reported alterations in the DAT level or function in SCZ patients (Artiges et al., 2017; Tseng et al., 2017; Lucarelli et al., 2019; Sekiguchi et al., 2019), but some of the results are still contradictory (Fusar-Poli and Meyer-Lindenberg, 2013). The described SCZ increase in DA synthesis/release in the rat dorsal striatum can be reproduced in preclinical models with alterations which resemble SCZ early symptoms (Petty et al., 2019). These general features are confirmed in a mouse model of NMDA receptor hypofunction in GABAergic neurons during development (Nakao et al., 2019), in mouse models studying SCZ genetic links to CACNA1C (Terrillion et al., 2017) and in Neuregulin 2 KO mice (Yan et al., 2018). Recent data managed to shed further light on the synaptic proteins involved in DA release, and how these are linked to SCZ by genetic studies. For instance both the somato-dendritic and axonal release of DA are controlled by RIM protein isoforms in the active zone and by the Rab3 counterpart *via* D2L receptors (Robinson et al., 2019). Glutamatergic effects on the DA release machinery are most likely indirect and sustained by GABAergic interneurons at least in cortical regions (Molinaro et al., 2015). In fact, antipsychotic agents do not completely manage DA synthesis/release alterations, even in presence of efficacy on psychotic symptoms (Wheeler et al., 2015; Weinstein, 2019).

### DR Subtypes

DR are integral membrane receptors coupled to G proteins (Beaulieu and Gainetdinov, 2011; Thal et al., 2018). The dopaminergic system signals through “D1-like” D1 and D5 receptor subtypes and “D2-like”: D2Short (S), D2Long (L), D3 and D4 receptor subtypes (Xin et al., 2019). There is some difference in the affinity of DA for D1-like receptors and D2-like receptors, mostly reported on the basis of receptor-ligand binding studies in recombinant systems (**Supplementary Material: Table 1**). D2-like receptors have a 10- to 100-fold greater affinity for DA than the D1-like family, suggesting that the balance of D2-like vs. D1-like receptor signaling can change depending on extracellular DA concentrations. A general view supports the specific engagement of D1 receptors in cortical regions when in presence of burst firing (Dreyer et al., 2010; Nair et al., 2014) while DA tonic activity affects only postsynaptic D2-like receptor signaling (Caravaggio et al., 2020). Differences in DR affinity may not be however the only relevant factor when discussing DR engagement in physiological conditions. The timescale of DR engagement (minutes) and the relative DR abundance in complex circuits need to also be taken into account (Hunger et al., 2020). The role of DR in different neuronal populations in striatum can be an example of this complexity. D1 and D2 receptors are generally segregated in striatal GABAergic medium spiny neurons (MSNs). D1-MSNs respond mostly to DA burst signals (Yapo et al., 2017), while optogenetic studies show that the effect of DA burst firing on D2 is not occluded by the presence of a background DA tone. D2-MSNs can therefore respond to a broader range of stimuli (Marcott et al., 2014). Cholinergic interneurons in the same region also receive an important DA/glutamate corelease input during burst firing. These cholinergic neurons express the receptor D5 (D1-like) responsible for an excitatory response after a bursts of DA release and D2-like receptors which trigger an hyperpolarization (a pause in the cholinergic signaling sequence) when activated. These events are in temporal sequence with the NMDA activation after glutamate/DA corelease creating a specific pattern of activity in these interneurons (Wieland et al., 2014). In the nucleus accumbens (nAcc) finally D1 and D2-like receptors work in cooperativity (heterodimers) in the same neuronal population and still a local complex coding of response to DA release fluctuations can support motivation and decisional processes (Hamid et al., 2016).

The original classification of DRs subtypes signaling mechanisms on the basis of cAMP stimulation and/or inhibition is no longer so useful given the substantial complexity of the heterocomplexes formed by DR. The DR - cAMP cascade is in any case directly linked to mRNA translation enhancement *via* PKA and serine-residues phosphorylation of ribosomal protein S6. So transcriptional - translational control can be considered a specific part of the DRs activation cascade. Only D1 and D2/D3 will be further discussed in this review as DR most involved in SCZ related alterations. D5 research did not produce convincing evidence so far of robust SCZ association (Hwang et al., 2012) and a link to stress and GABA transmission is the only new element of relevance for D4 in SCZ psychosis (Tan et al., 2019).

### D1 Receptors

When discussing D1 in the context of SCZ, the most important aspects are certainly related to the prefrontal cortex (PFCx) regions and the cognitive deficits observed during the disease (Arnsten et al., 2017). D1 activates a postsynaptic Gs/Golf protein complex with a final increase in intracellular cAMP levels. PDE1b is the most relevant enzyme for the cAMP degradation upon D1 activation (Yamamoto et al., 2013; Yano et al., 2018). Two cAMP sensors link D1 activation to the ERK cascade: PKA and NCS-RAP/GEF2. Both proteins are important to trigger neuroplasticity effects (Jiang et al., 2017). Prolonged agonist activation of the D1 receptor leads to phosphorylation of the intracellular domains by G protein coupled receptor (GPCR) serine and threonine kinases (GRKs) and other kinases like GSK3b. They trigger the translocation and coupling of β-arrestins and D1 receptor endocytosis (Wang et al., 2017). The scaffolding function of β-arrestins enables the gathering of various other signaling components (cAMP independent). D1/D3 heterocomplexes transactivation can also switch D1 signal toward a cAMP independent cascade (Guitart et al., 2019). D1 has been the focus of past SCZ research because of its functional role in the potentiation of postsynaptic NMDA currents *via* a receptor complex with NR1a/NR2a including PSD95 (Zhang et al., 2009; Desai et al., 2017). D1 activation triggers NR1-CaMKII coupling and enhancement of CaMKII activity; mGlu5 phosphorylation by MAPK and potentiation of the effect of Pin1 - Homer1 (Nai et al., 2010). A multicompartment model of this control in striatal medium spiny neurons (MSN) involves STEP tyrosine phosphatase (Beutler et al., 2011; Gutierrez-Arenas et al., 2014). The D1-dependent engagement of Fyn kinase leads to an enhancement of NMDA NR2b subunit channel activity also of specific relevance in MSN in striatum (Hu et al., 2010) NMDA – D1 interplay *via* Fyn kinase could be also more broadly relevant across glutamatergic synapsis in cortical regions given the long term effect on the function of ELF2 (David et al., 2020). A more downstream control on the same path can be made *via* PKA activation and by PDE10 inhibitors and similar considerations can be applied to D2 intracellular cascade in MSN (Nishi et al., 2011; Harada et al., 2020). D1 may be present in heterologous glutamatergic pre-synapsis possibly in heterocomplexes (D3)? in prefrontal cortex and hippocampus with an effect on glutamate release (Hikima et al., 2016).

### D2/D3 Receptors

D2-like receptors (D2/D3) are the main targets of antipsychotics (Zhang et al., 2020). The D2 receptor is present in two isoforms D2S and D2L which differ because of a 29 AA insertion in the third intracellular loop on D2L (Zuk et al., 2020). Both receptors can inhibit intracellular cAMP *via* Gi. The inhibitory effect of D2 (and D3) on membrane excitability is generally due to the coupling to GIRK channels *via* Go (Kv 1.1, 1.2, or 1.6 - possibly Kv3) (Huang et al., 2013; Bonifazi et al., 2019). Both D2S/L receptors can initiate a cAMP-independent pathway by promoting the association of a signaling complex containing AKT1, PP2A, and β-arrestins leading to the activation of both ERK1/2 and GSK3b signals (Chen et al., 2016). The D2 receptor establishes a complex with DISC-1 that facilitates GSK3 mediated signaling and inhibits D2 agonist mediated receptor internalization, further enhancing the final D2 mediated effects (Su et al., 2014). Antipsychotics seem to be able to uncouple this complex (Zheng et al., 2019). The D2S is dominant in the cell bodies and projection axons of the dopaminergic cells in mesencephalon, while the D2L is a mainly postsynaptic receptor strongly expressed by neurons in the striatum and nAcc, brain structures targeted by DA terminals. In cell types of relevance for SCZ like MSN or cortical pyramidal neurons, D2L is able to trigger PKA activation possibly because of receptor transactivation (Castellani et al., 2017). DARPP32, RCS, and ARPP16 are the most important PKA targets of the D2 effects (Walaas et al., 2011). D2L activation can also recruit c-Src to transactivate the PDGF receptor and downstream Ras/Raf/MEK/ERK signaling cascade. This pathway represents a main stimulus for dendritic formation in striato-pallidal MSN (Shioda et al., 2017). D2S auto-receptors (on dendrites and soma) are known to inhibits cell firing, activate DA reuptake and inhibit DA synthesis. The work of Purves-Tyson confirms that D2S, VMAT2, and DAT mRNAs are significantly decreased in schizophrenia, with no change in *DRD3* mRNAs, and DAT protein between groups (Purves-Tyson et al., 2017). Other studies have verified that these alterations are sensitive to stress (Sallis et al., 2020) and present in drug-naïve SCZ patients not previously treated with antipsychotics (Tseng et al., 2018). In the same presynaptic compartment D2S can inhibit the trace amine receptor TAAR1 with a final potentiating effect on the DA release in striatum (Leo et al., 2014; Su et al., 2014). The distribution of TAAR1 is predominantly intracellular thus being uniquely positioned to regulate aminergic activity (possibly including DAT function) (Asif-Malik et al., 2017). The recent positive clinical results obtained with the TAAR1 agonist SEP-363856 tested as antipsychotic provide a confirmation of the relevance of the observed alterations in presynaptic DA release in SCZ (Pei et al., 2016; Koblan et al., 2020).

**Figure 1:**
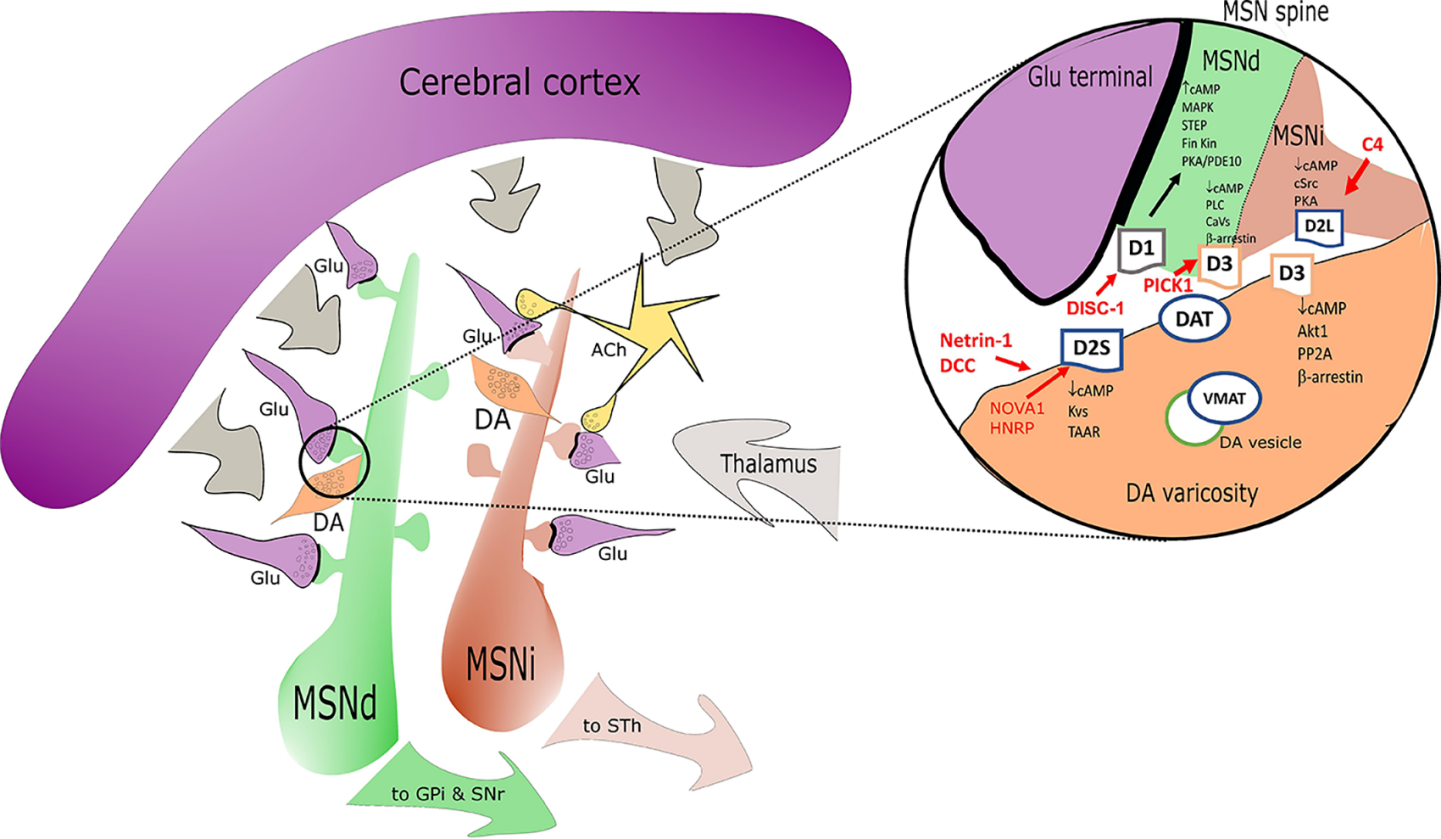
Simplified sketch of the dopamine receptors (DR) connectome in the basal ganglia/striatum with a zoom (right circle) on signal transduction at presynaptic level in medium spiny neurons (MSN) dendritic boutons. Highlights on the elements associated with SCZ alterations are depicted in red. D1 positive medium spiny neurons of the direct pathway (MSNd) are in green, inhibitory D2 positive MSN of the indirect pathway (MSNi) are in red. Glutamatergic cortical input - presynaptic terminals are in magenta. DA “en passant” boutons are indicated in orange and in close proximity of glutamatergic postsynaptic spines. Cholinergic interneurons are in yellow. In the magnification on the right note the distribution of DR: D2s and D3 are presynaptic in DA terminals; D1/D3 postsynaptic in MSNd and D2L postsynaptic in MSNi. Other projections are in gray. Abbreviations: ACh, acetylcholine; DA, dopamine; Glu, glutamate; MSNd/i, direct/ indirect path projecting MSN neurons; GPi, internal segment of globus pallidus; SNr, substantia nigra, reticular part; STh, subthalamic nucleus; other common abbreviation and protein names as cited in text.

The D3 receptor is efficiently coupled to Gi/o at pre- and postsynaptic sites and in cell bodies. Some D3 intracellular pathways are similar to those observed for D2 (Guitart et al., 2019). The D3 receptor can however be sequestered in an inactive state at the membrane level rather than internalized (Zhang et al., 2012; Zhang et al., 2016; Zheng et al., 2016). D3 can work in complex with D1 receptor and thanks to this, D3 agonists can stimulate cAMP production and even GABA release. This D1/D3 interaction also facilitates non cAMP related intracellular signaling as demonstrated with biased ligands (Guitart et al., 2019) (see section 3). At postsynaptic level in MSN, D3 modulates Ca^2+^ channels *via* PLC and PP2B. At extra-synaptic location (cell bodies) D3 receptors have been reported to selectively modulate Ca^2+^ influx through low-voltage activated (Ca_V_3, T-type) Ca^2+^ channels, in a β-arrestin-dependent mechanism. In other cases, non-canonical DR mediated events like the D3 interaction with the ghrelin receptor need to be invoked (in hippocampus) to explain a final effect *via* Galphaq-PLC-IP3-Ca^2+^ (Kern et al., 2015). The D3 receptor is able to interact with nicotinic receptors (for instance alpha 4 containing nicotinic receptors) in particular in VTA (Bontempi et al., 2017) and represents a main point of cross talk with the cholinergic system (Matera et al., 2019). D3 turnover is controlled by the EGFR tyrosine kinase signaling cascade (Zhang et al., 2020). EGFR phosphorylates GRK2 which then phosphorylates the intracellular domain of the D3 receptor to trigger D3 intracellular receptor degradation (Sun et al., 2018). PICK1 instead seems to be able to control surface D3 levels. PICK1 is present in dopaminergic neurons in close proximity with D3 (also D2 and DAT) at cytosolic level and an increase in PICK1 lowers the surface density of D3 (Zheng et al., 2016). D3 effects can be increased in presence of NMDA receptor hypofunction. Upon NMDA activation CaMKII alpha is recruited to D3 by rising Ca^2+^ to increase the CaMKII alpha-mediated phosphorylation of D3, thereby transiently inhibiting D3 efficacy (Liu et al., 2009). This CAMKII control on DA/NMDA interplay is potentially very relevant in SCZ and core to the therapeutic interventions required to limit D3 overactivation. See **Figure 1** for DR and signal transduction at synaptic level.

### DR Dimerization and Complexes

As for many GPCRs, all DR subtypes form homo and heterodimers *in vivo* with effects on native receptors signaling. DR dimerization involves transmembrane domains 5 and 6. This interaction can be a transient process, stabilized in presence of agonists like dopamine or quinpirole (Kasai et al., 2018) and it is of potential pathophysiological significance for SCZ. The balance of D2 homodimers to monomers has been also associated to amphetamine sensitization in animals, a further element related to SCZ (Weidenauer et al., 2020). This is why the generation of bivalent DR ligands has been attempted by several groups (Carli et al., 2018). The most common DR heterodimers/tetramers observed *in vivo* are D1/D2, D1/D3, D1/H3 and D2/A2A (Borroto-Escuela and Fuxe, 2019). They all affect the MAPK response of these receptor systems, D1/D3 also modify recruitment of β-Arrestin-1 and heterodimer internalization. mGlu5/D2, D2/mu opioid receptor, D2/neurotensin 1 receptor, and D2/5-HT1a heterodimers have been also described, but not necessarily in the context of SCZ (Lukasiewicz et al., 2016; Qian et al., 2018a; Qian et al., 2018b). They can all be potentially relevant for the effects of antipsychotic agents and for the generation of new ligands with unique pharmacological properties (Hubner et al., 2016). A different type of interaction has been described for D1 and NMDA receptors. In this case the presence of a membrane cluster in hippocampal neurons has been convincingly demonstrated during the past decade (Ladepeche et al., 2013). D1 activation is associated with increased NMDA trafficking to the synaptic surface and vice-versa. The proposed model shows D1 receptors dynamically retained in clusters in the vicinity of glutamate synapses where they interact with NMDAR. DR activation disrupts this interaction and favors the lateral redistribution of both receptors. D1Rs moves to extra-synaptic areas, whereas NMDA receptor reaches the glutamatergic postsynaptic density. Most importantly anti-NMDA antibodies from SCZ patients disrupt NMDA trafficking and reduce D1 trafficking as well. A region contained in the intracellular C-terminus of the D1 receptor is involved in this interaction with the NMDA receptor (Grea et al., 2019). More complex structures are also reported in the cortex involving D1, H3 and NMDA receptors (Rodriguez-Ruiz et al., 2017).

### DR Turnover

Palmitoylation at the C-terminus of the DR protein has been documented for D1, D2, and D3 receptors as reversible switch for DR signaling *via* the cAMP path (Ebersole et al., 2015; Arango-Lievano et al., 2016). The most important posttranscriptional modification of D2 and D3 receptors is the N-linked glycosylation that classically affects both correct cell surface expression and signaling/internalization (caveolin - chlatrin mediated) (Min et al., 2015). D1 and D2 are localized to different endocytic vesicles after internalization. D1 is recycled back to the cell surface in a process controlled by the VPS35 complex (Wang et al., 2016), while prolonged agonist stimulation causes D2 trafficking into lysosomes and subsequent receptor degradation by a Rab5 GTPase controlled pathway (Shioda et al., 2017; Shioda, 2017). A specific presynaptic control on D2S membrane density is exerted by the L1 close homolog adhesion factor (also a risk gene for SCZ) (Kotarska et al., 2020). Presynaptic D2S receptor density is directly or indirectly affected by ALK and possible transactivation mechanisms (He and Lasek, 2020). The overall complexity of the control of D2 receptor internalization (vs D3) is possibly justified by the major biological role of D2 surface density adjustments, required in different circuits depending on DA content. A specific example is the D2 vs D3 relative control by Dysbindin 1 (Leggio et al., 2019). Dysbindin (SCZ risk gene associated with cognitive symptoms) is mainly expressed in hippocampus and dorsolateral (DL) PFCx. It is a component of the multi-subunit complex BLOC-1 where it interacts directly with MUTED (also probably associated with SCZ). Both dysbindin and MUTED siRNAs increase cell surface D2 receptors and block DA-induced D2 internalization in human and rat cells. Dysbindin variants are known to modify the cognitive response to antipsychotics. This effect is most likely related to the parallel Dys1/D3 signal reduction that favors a D2 component in cortical regions (Leggio et al., 2019).

Other types of control on DR density are exerted at source at the transcriptional level. A recent analysis of proteasome alterations in SCZ points at spliceosome nuclear protein and calmodulin related pathways. The control on the splice variants of the D2 receptor is exerted by NOVA1 and HNRP (Min et al., 2015), and D2 mRNA 3´UTR binding of microRNAs mir-9 and mir 328 inhibits messenger translation (Shi et al., 2014). Development mechanisms are directly impacting on DR expression. In particular DISC-1 can translocate with KLF16 into the nucleus and recruit SIN3A corepressor to the D1 locus (Suh et al., 2019). The DISC-1 related complex is a main hub that could bring more specific information on SCZ developmental aspects in terms of consecutive development related alterations in glutamatergic (NMDA/AMPA) and dopaminergic responses (D1+D2+D3) in key SCZ regions like dorso-lateral PFCx and the striatum (Onishi et al., 2018; Jacobi et al., 2019). The expression control can also be exerted more dynamically on the D1 intracellular signal transducers by nuclear receptors like Nr4A1 (Nurr77) (Cirnaru et al., 2019). Another nuclear factor involved in shaping dopaminergic terminals is Nurr1, highly relevant for the D2 receptor network and its circadian cycling (Chung et al., 2014; Torretta et al., 2020). See **Table 1** supplementary material for a summary. Until puberty, the DA system maturation is controlled by the netrin-receptor DCC mediated organization of DA neurons in the meso-cortical limbic system and the projections to PFCx (Vosberg et al., 2020). Axon navigation is directed by extracellular axon guidance cues, which induce molecular changes in the axonal growth cones in response to extracellular levels of DA (via D1 in complex). The DCC gene keeps being a confirmed SCZ genetic link across several studies (Vosberg et al., 2020) with a particular effect on the anatomical connectivity of the nigra/VTA dopaminergic pathways and the final distribution and relative density of DR. In animal models, SCZ-like symptoms seem to correlate with netrin 1 - DCC related alterations in size, complexity and density of DA spines (medial PFCx layer V pyramidal neurons). Other genetic SCZ links (for example RGS12) concur on DA synthesis and release (Gross et al., 2018; Kos et al., 2018). A common upstream element affecting the expression of D2, COMT and structural proteins at presynaptic DA level is the zinc finger element ZFN804A (Girgenti et al., 2012), coded by another SCZ risk gene (Zhou et al., 2020).

## Section 2: DR Alterations in Schizophrenia

The current understanding of the role of DR in SCZ is in full expansion, thanks to developmental brain studies and the advancements of imaging techniques. DR expression is segregated across neuronal populations and associated with temporal and coupling differences in activation properties. This distribution is respected in SCZ, while a variety of DR β-arrestin mediated intracellular signaling show clear alterations in SCZ disease models. Some developmental and connectivity aspects of DR distribution are maintained across species and useful for the definition of SCZ as a developmental disease across circuits (Sonnenschein and Grace, 2020).

### Prefrontal Cortex Neurocircuit(s) Affected by SCZ and DR

Connectivity measures across different SCZ studies are not always easy to compare, but some key elements are constant across patient groups, detection modalities and data interpretation: the involvement of striatal-thalamic and PFCx connections in SCZ (Zhao et al., 2020). Imaging, functional and circadian studies are also in general agreement on the presence of main alterations in the PFCx of SCZ patients, in particular dorso-lateral and cingulate regions (Seney et al., 2019). PFCx circuits are central to cognitive functions and linked to the different aspects of cognitive deficits and positive symptoms as observed in SCZ. Dorso-lateral PFCx weaker processing of sensory information from thalamus is in fact associated with hallucination experiences which are common in > 50% of the SCZ patients (Daskalakis et al., 2020). The molecular studies point at parvalbumin positive (PV+) GABAergic interneurons and cortical pyramidal cells networks as both altered in SCZ PFCx and across species in SCZ models (Chung et al., 2018; Petralia et al., 2020; Wang et al., 2020; Weidenauer et al., 2020). Dopaminergic ascending terminals reaching these neurons are also hypofunctional (Rao et al., 2019). Dopamine release enables the PFCx to compute and generate spatio-temporally diverse and specialized outputs, but these are not a linear function of the DA release input. Thus, it is quite complex to establish the functional correlates for cortical functions. Rapid, transient changes in DA transmission in PFCx are observed in response to task events, such as cues and rewards whereas prolonged responses are relevant to emotional states and motivation (Lohani et al., 2019). DA neurons in the region are mainly coming from the VTA and the terminal density in PFCx is much lower (in terms of DAT content) when compared to the striatal regions.

D1 receptors are enriched in pyramidal cells in both layers 5 (thin-tufted layer) and 6 projecting in turn to contralateral cortex, striatum, and claustrum. D1 receptors are also present in interneurons and enriched in a specific population of VIP+ calretinin positive interneurons (Anastasiades et al., 2019; Saffari et al., 2019). D1 receptors strongly enhance action potential firing in this subset of cortico-cortical neurons and VIP+ interneurons and the modulation *via* D1 receptors can influence both excitatory and disinhibitory microcircuits in the PFCx (Anastasiades et al., 2019). This PV+ interneuron circuits are a the main point of interaction between mGlu5/NMDA and D1 (D2-like) receptors, both involved in the control of the glutamatergic input from pyramidal cells (Nicoletti et al., 2019). D1 is important for the correct migration of the dopaminergic terminals which increase throughout adolescence across species. Developmental studies in netrin-1 receptor DCC deficient mice demonstrate a role for DA in adolescent brain axon growth. DCC controls in fact the extent of this protracted growth by determining where and when DA acts. Pyramidal neuron morphology studies and cognitive performances show that the lack of DCC causes dopaminergic deficit across PFCx and morphological changes in pyramidal neurons (Reynolds et al., 2018). This process can be influenced by stress. The DA deficit in PFCx regions following this hypothesis may be then of developmental origin and caused by morphological alterations affecting DA terminals, pyramidal cells and interneurons.

D2/3 receptors are also differentially expressed in PFCx and their activation contribute to specific cognitive processes (Robinson and Sohal, 2017; Bailey et al., 2020; Papenberg et al., 2020). D2 are enriched within subcortically projecting L5 pyramidal neurons thick-tufted pyramidal cells, with projections to thalamus and pons, but not contralateral cortex (Yu et al., 2019). These neurons exhibit a prominent hyperpolarization-activated cationic current. In this population, pharmacological activation of D2 elicits a profound after depolarization that only occurs when NMDA receptors are coactivated. D2 signal in this case is triggering a Gs- cAMP/PKA pathway in a non-canonic manner (Robinson and Sohal, 2017). D2 are also expressed in PV+ interneurons, a property acquired during adolescent brain maturation (Urs et al., 2016). The D2 network controls the connection to the hippocampal system (Tomasella et al., 2018; Khlghatyan et al., 2019). Species related differences in this circuitry could be large, so human data are needed for the correct interpretation of the results (Gonzalez-Burgos et al., 2019). The cortical D2 mediated effects of the most common antipsychotics (antagonists and partial agonists) have been extensively evaluated. This is mostly because these agents cannot rescue the cognitive impairment associated with schizophrenia, with possibly few exceptions (amisulpride or 5-HT1A partial agonists) (Park et al., 2019; Huang et al., 2020).

D3 are expressed by a distinct population of prefrontal neurons and they also represent the main auto-receptor controlling DA release in prefrontal cortex. D3 expression defines an additional class of L5 pyramidal cells that largely lack D1 or D2 coexpression. L5 D3-expressing neurons are similar to D1-expressing cells in their synaptic connectivity, with projections to contralateral cortex. D3-expressing neurons could be distinguished from D1- or D2-expressing neurons by dendritic morphology, intrinsic electro-physiological properties and by the manner in which DA regulates neuronal function. In these neurons in fact D3 selectively regulates the dynamics of voltage-gated calcium channels localized to the site of action potential initiation in the axon initial segment, with a marked suppression in the generation of high-frequency action potential bursts. D3 regulates Ca_V_3.2 channels through a non-canonical, arrestin-dependent pathway. The D3 plays therefore a unique role in the regulation of pyramidal cell excitability (Clarkson et al., 2017). The D3 receptor function has received attention because it could be a discriminant of the clinical effect of different antipsychotics (Girgis et al., 2020) and because of the potential to address SCZ negative symptoms. In fact, D3 are associated to a cortical circuit important for all the different SCZ symptoms. The D3 controlled PFCx projections to hippocampus are interesting in this sense (Provenzano et al., 2020). The recent paper from Meier et al. shows the effect of a preferential D3 partial agonist Cariprazine on gamma oscillations in hippocampal slides further supporting the general assumption that gamma waves could predict psychosis and *in vitro* NMDA hypofunction, and that D3 functional reduction can stabilize the alterations of the signal caused by NMDA hypofunction (Meier et al., 2020). Treatment response to antipsychotics may be predicted looking at the effect on hippocampal- cortical connections and again these changes could be in part D3 related (Guma et al., 2019; Blessing et al., 2020). The observed hippocampal alterations in some SCZ patients (psychotic) also support the presence of hippocampal immaturity at least in a subgroup of SCZ patients (Alvarez et al., 2020; Cachia et al., 2020). There is therefore a renewed interest for the hippocampal models in SCZ, because it is possible to study developmental changes which are closer to those observed in man and because it is easier to obtain NMDA receptor hypofunction (Alvarez et al., 2020). In a mouse model of postnatal NMDA hypofunction (NR1a KO) the effect seems to be selectively associated with PV+ interneurons (in cortex and hippocampus among other areas). In this animal model both cortical hypo- and striatal hyperdopaminergic phenotypes can be observed (Nakao et al., 2019). The reason(s) behind these extensive dopaminergic changes across areas are still not fully understood, but SCZ genetic data related to ancillary proteins for the NMDA receptor function also support this hypothesis. Very recent work has also given renewed attention to circuit(s) involving PFCx areas like DL or the orbitofrontal (and cerebellum) in relation to some aspects of negative symptoms in SCZ (Walton et al., 2018; Brady et al., 2019). It is possibly too early to include a conclusive map of DR expression in within these pathways. The DISC-1 developmental mouse model could however help to analyze these circuit(s), considering the main impairment observed in sociability measures (Sultana and Lee, 2020). The PV+ interneurons can also be a starting point to address the network in terms of developmental changes. Recent DISC-1 studies report a reduction of spontaneous inhibitory transmission onto L2/3 PV+ interneurons in medial PFCx and a decreased feed forward inhibition onto L2/3 pyramidal neurons (Delevich et al., 2020).

### Striatal Circuits Alteration(s) in SCZ and DR

The main role of the striatum is the integration of cortical and thalamic glutamatergic projections (Hunnicutt et al., 2016; McCutcheon et al., 2019). The striatum is at the center of a DA-sensitive basal ganglia circuit associated with psychosis, SCZ related motor dysfunctions and reward deficits. A summary of all the direct and indirect evidences of striatal DA alterations in SCZ was recently published (McCutcheon et al., 2020). All data confirm the presence of presynaptic DA sensitization and elevated DA synthesis and release capacity (Brugger et al., 2020; Weidenauer et al., 2020). Higher striatal DA synthesis and higher DA release correlated with worsening of psychotic symptoms in SCZ patients and were also supported by neuromelanin observation (Weinstein et al., 2017). Excess striatal DA in SCZ is not related to changes in DA innervation (Wengler et al., 2020). There have been extensive efforts to describe the neuroanatomy of striatum, and the cellular distribution of DR (Soares-Cunha et al., 2016; Clarkson et al., 2017). Substantia nigra DA projections mainly reach the dorsal striatum (Uchigashima et al., 2016) while ventral tegmental area (VTA) projections from the mesencephalon reach the ventral striatum (nAcc). Striatal neurons that receive DA inputs are mainly GABAergic medium spiny neurons (MSN). MSN neurons are the recipients of both DA and glutamatergic (from PFCx and thalamus) projections, they represent therefore a core neuronal element for both DA and NMDA hypothesis in SCZ. The MSN projecting to the internal segment of globus pallidus/nigra pars reticulata express D1 receptors, while those projecting to the external segment of globus pallidus are essentially expressing the D2 receptors. The two types of neurons are finely intermingled across the whole striatum (Ren et al., 2017). There is also a not so small population of MSN that express both D1 and D2 receptors. They are usually described as enkephalin receptor positive neurons, they express specifically the subunit GluA3 of the AMPA receptor and project broadly to nuclei containing DA neurons cell bodies, to the nAcc and the ento-peduncular nucleus among others (Perreault et al., 2011). The cross talk of interneurons at this level is a main filter on the cortical input. Clearly, different DR contribute to the final effect, depending on receptor distribution across different types of interneurons (Burke et al., 2018). For example the D1 activity in MSN is inhibited by the cholinergic tonus (M4 mediated) (Nair et al., 2019). In SCZ increased spine density have been observed in dorsal striatum MSN. Converging evidences suggest a critical role of the dopaminergic system in adapting synaptic plasticity of glutamatergic inputs (synaptic spines). Early in development, the DA system has fundamental roles in forebrain differentiation and circuit formation (Brignani and Pasterkamp, 2017), but DA tone also has clear effects on glutamatergic spine density at adult stage. It is however not clear how SCZ specific NMDA alterations could impact on the system. The recent and seminal work of the group of Prof. Groc, using single molecule-based imaging shows that NMDA antibodies present in some SCZ patients with psychotic symptoms are specifically changing the surface dynamics and nanoscale organization of synaptic NMDA and its anchoring partner the EphrinB2 receptor in synaptic spines in hippocampal neurons, ultimately preventing LTP potentiation (Jezequel et al., 2017; Jezequel et al., 2018). As expected this causes a small reduction of the D1 surface expression in the same cellular system (Grea et al., 2019). The associated intracellular DA signaling effects however could be more deeply modified because of this lack of NMDA/D1 interaction. It would be equally important to study these NMDA-antibody related changes in the context of the striatal circuits in particular on MSN D1 mediated signal and during development. The D1 receptor in dorsal striatum has been also involved in the sensorimotor gating alterations observed in SCZ but these mechanisms needs to be verified in man and with selective agents given the main differences in anatomical connectivity (Aguilar et al., 2018).

### Striatal D2/D3 Receptors and SCZ

There are main differences in the DA input across the different striatal regions. This is particularly true for the D2 receptor function across dorsal striatum and nAcc. Increased DA D2 sensitivity in the nAcc is related to differences in coupling to Go vs. Gi (Marcott et al., 2018). The striatal D2 related control on reward is a key aspect of the effects of antipsychotics. Psychotic symptoms have been in fact linked to salience changes in the reward system circuit and blocking D2 controls psychotic symptoms including a normalization on reward disturbances (Han et al., 2020). A direct relationship between D2 receptor blockade, normalization of reward processing and symptom improvement was recently further supported by a small study in antipsychotic-naive first-episode SCZ patients (Wulff et al., 2020). Cognitive flexibility (reversal learning) is another aspect of D1/D2 related deficits that is linked to DA striatal functional regional differences (Sala-Bayo et al., 2020). The cellular basis of the role of striatal D1 vs. D2 in reward and learning have been further clarified by the work of Iino et al., 2020, showing in rodents the presence of a D2 controlled spine plasticity in MSN, that can be reversed with a D2 antagonist (Iino et al., 2020).

D2 antagonism is still recognized as a main stay of SCZ therapy and the D2 receptor is considered to be directly or indirectly responsible for the efficacy of the majority of typical and atypical antipsychotics. This is coherent with the general observation of a main role of DA control of cortico-striatal synchronization of D2-MSN neurons (via D2-GPRIN AKT) (Karadurmus et al., 2019). The tetra complex A2A-D2 receptors (plus AC5) is really central to multiple effects of both adenosine and DR ligands in the striatal region (Ferre et al., 2018; Bonifazi et al., 2019). mGlu5 receptor can be also included in a complex interaction with D2-A2A in GABAergic neuronal terminals providing a multiple way to increase GABA release (Borroto-Escuela et al., 2016; Sahlholm et al., 2018). It is becoming therefore apparent that D2 receptor function is heterogeneous and possibly strictly dependent on the neuronal type expressing the receptor in different cortical and sub-cortical regions. Considering the role of D2 receptor in the control of emotional, cognitive and sensory functions alterations in SCZ it is therefore important to revisit the molecular aspects of this receptor and possibly even the pharmacology of the different antipsychotics (Quintana and Beaulieu, 2019). For instance the D1/D2 complex (possibly) present in some MSN exhibits the remarkable property of a coupling to a Gq- PLC mediated increase in intracellular calcium release and CAMKII phosphorylation (Perreault et al., 2011). This complex may represent an interesting new pharmacological target in SCZ. The D2S receptor is involved whenever SCZ treatment resistance is discussed or phenomena of presynaptic D2 receptor supra-sensitivity induced by antipsychotics (Amato et al., 2019).

Motivational deficits in SCZ are most likely associated with cortico-striatal circuits involving the VTA, and the ventral striatum (Aberg et al., 2020; Kontaris et al., 2020). Clinical observation keep suggesting some involvement of ventral striatum in the control of motivation, emotions and social behavior as relevant for negative symptoms in SCZ with regular debates on the matter (Fareri et al., 2017; Stepien et al., 2018; Waltz et al., 2018). Interestingly, D3 receptor expression is enriched in midbrain ventral striatum (including nAcc) (Slifstein et al., 2020) where the receptor is present on pre- and postsynaptic locations and can also work in cooperation with the receptor D1 (in MSN - AKT signal) (Castrellon et al., 2019; Guitart et al., 2019). The D3 receptor has been linked to control of DA firing in VTA, emotion and reward control in animal models (Takeuchi et al., 2019), but the lack of selective D3 ligands has so far hampered specific research on the subject (Correll and Schooler, 2020). Cholinergic interneurons in the ventral striatum, particularly those in the insula major of Calleja are highly enriched in D3 receptor, making these cells extremely sensitive to DA from VTA projections. Also in this case a D1/D3 complex is probably present. In this region as well as in cerebellum or other extra-striatal circuits, the D3 receptor has been linked to thermoregulation and sleep/wakefulness, which are potentially relevant for the control of some aspects of SCZ (Luo et al., 2018). Calleja islands are also a site related to adult neurogenesis in ventral striatum across species: these neurons are D3, Erb4 and neuroregulin1 positive.

## Section 3. DR Ligands and SCZ Therapies. The New Wave of Ligands With Potential Relevance for Therapy or Brain Imaging

The discovery that DA effective drugs for treating SCZ is redeemable to the elegant work of Carlsson and Lindqvist in the early 60’s and to the identification, a decade later, of the antipsychotics/DA receptor. Atypical antipsychotics developed in the 70’s and 80’s, included serotoninergic complementary mechanisms, as observed with clozapine, the prototypical atypical antipsychotic, to improve treatment compliance (Aringhieri et al., 2018). Historical perspectives on SCZ drugs generally highlight the DA receptor D_2_ antagonism as main mechanism of action (Madras, 2013), but the pharmacology of antipsychotics is much more complex and requires a specific discussion on DR selectivity and serotonin receptor poly-pharmacology (Butini et al., 2016; Aringhieri et al., 2018; Moritz et al., 2018; Bueschbell et al., 2019). Important discoveries were made in the DA field during the past decade, in particular in relation to the pharmacology of DR ligands. DR heterodimers have been described in different brain regions and used to explain the complex biological effects associated with DR activation (Borroto-Escuela et al., 2018). Exciting data from crystallographic studies have supported a wave of drug discovery projects looking for new antipsychotics (Chien et al., 2010; Wang et al., 2017; Wang et al., 2018). DR signaling versatility is further magnified by context dependent dissecting signatures or “bias” (Urs et al., 2017) extending the potential for optimized pharmacological interventions. It is possible for instance to separate β-arrestin mediated signals using biased D1 agonists (Urs et al., 2011; Gray et al., 2018). Several recent contributions are available on this matter (Vyas et al., 2020). The potential therapeutic applications of biased D2 ligands to new SCZ therapies, has fuelled new interest on D2S vs. D2L or cAMP independent intracellular pathways, looking for agents with less motor side effects. D2 β-arrestin-biased ligands are now available (Park et al., 2016) and they may provide some pharmacological advantages, at least on the basis of the results in preclinical models (Urs et al., 2017). These agents are not per se D2 selective since they also interact with the D3 receptor and might require the presence of an heteromeric complex with the receptor A2a for the final effect. There is therefore a need for a different look at DR ligands pharmacology *in vitro*. We should possibly reconsider aspects like receptor internalization or intracellular recycling also for the main active metabolites or when comparing antagonists and partial agonists (De Vries et al., 2019). See **Table 2 Supplementary Material** for chemical series of DR ligands and representative compounds described in section 3.

### DR Ligand Receptor Interactions

The most interesting finding in the field of DR is certainly the crystal structure of D2, D3. and D4 receptors and how this was used to identify new series or new mechanisms of ligand receptor interaction. Homology models are also extremely helpful for D1 and D5 with some main limitation for specific domains with reduced identity (Bueschbell et al., 2019). The DA binding site is contained in a membrane pocket formed by the TM3/5/6/7 with similarities across biogenic amines GPCRs. Molecular docking studies for the D1 receptor were able to demonstrate the presence of allosteric sites that were further targeted to obtain highly selective positive allosteric modulators with high potency, weak agonist properties and able to increase DA response (cAMP) (Bruns et al., 2018). The mode of interaction of biased agonists is different since they fail to trigger D1 receptor desensitization *in vitro*. The current model supposes a docking in within the DA site, but with differences in interactions with TM3/5 and extracellular loop 2 (Gray et al., 2018). The rapid advance of the pharmacology of D1 receptors bringing new drugs to the clinic is a clear demonstration of the therapeutic impact of research on DR-ligand interactions (Hall et al., 2019). For D2/D3 biased ligands the drug design is complicated by the needed poly-pharmacology vs. 5-HT1A or 5-HT2A receptors which contribute to the clinical efficacy and also is intrinsic to some pharmacophore (Ma et al., 2019). The ligands cocrystallized in the different D2/D3 studies are haloperidol, risperidone, nemonapride and eticlopride, non-selective but potent antagonists (Fan et al., 2020). Thus no main difference was expected. In reality the results show differences in D2 inactive conformation that suggest different receptor inactive states (Lane et al., 2020). In addition the agonist binding pocket in the D2 allows an extension that has been used to study D2 > D3 and D4 selectivity (with agonist ligands) and to determine the possibility to obtain biased agonists for D2 (Fan et al., 2020). The re-assessment of the D2 interaction profile of different classes of D2 antagonists is also on the way (Zieba et al., 2019). The case of D3 is complementing this picture given the variety of new ligands currently available. Subtype-selective compounds have been sought for more than two decades with difficulties achieving sufficient selectivity and central exposure. Clinical PET data have recently provided encouraging results with cariprazine and F17464 (Slifstein et al., 2020). More recent D3 over D2 new ligands have been obtained exploiting the presence of a secondary allosteric D3 pocket to generate bitopic ligands with long molecular bridges. This strategy has allowed a powerful expansion in chemical possibilities even while maintaining the capacity to generate agents with biased activities (Rossi et al., 2017; Bonifazi et al., 2019). The concept of bitopic ligands is associated with the presence of two separated regions of the receptor with different vectors relevant for the affinity and the allosteric pocket interaction (usually driving D3/D2 selectivity considerations). Shorter D3 ligands will necessarily reside instead only in within the orthosteric pocket. Some interesting caged ligands for the D2/D3 orthosteric pocket could possibly help further pharmacological studies on this subject in native systems (Gienger et al., 2020). There is a second interesting aspect in the pharmacology of D3 bitopic ligands. It has allowed to show the presence of an alternative mechanism of D3 receptor internalization independent of β-arrestin and used by group II GPCR (Xu et al., 2019). Considering the excess D2 homodimers detected in schizophrenia (Wang et al., 2010), the effects of DA antagonists on these entities has been specifically explored using bivalent ligands (Pulido et al., 2018; Wouters et al., 2019). A molecular model of the homodimer has been also generated for D2 to provide docking information relative to bivalent ligands with different pharmacological properties (for example orthosteric and allosteric agents) (Kaczor et al., 2016). Other DR heterodimers were also considered as selective targets for this type of ligands (Carli et al., 2018), mainly because the differential expression of these dimeric receptor entities may allow a more precise approach to specific brain structures and pathways (Cortes et al., 2016; Foster and Conn, 2017).

### DR-Ligand Interaction Dynamics and Efficacy Studies

There are classic aspects of receptor pharmacology like constitutive activity or equilibria across receptor conformations which are quite difficult to address with DR, in particular when considering heterocomplexes. It should be however possible to better distinguish antagonists from partial agonists and systematically discuss on and off rates vs. affinity measures when presenting new DR ligands. Species specific differences are also seldom acknowledged. This systematic pharmacological work is required to make sense of the complex *in vivo* pharmacology of DR ligands (in particular D2/D3) also for antipsychotics already on the market. The case of D2 and D3 receptors is indeed quite interesting in this sense because of the complexity of the structure/activity database required to select new candidates and validate efficacy in comparison to reference antipsychotics. Several groups have generated a variety of synthetic ligands concurring to build similar molecular models including dynamic aspects of DR receptor activation over time. In recombinant systems at least, we witness some amazing activity switches between agonist and “antagonist” properties across different series that require further dynamic considerations (Tan et al., 2020). Destabilization of D3 inactive state(s) and flexibility of the ligands are among the elements that the most recent model available is proposing (Ferraro et al., 2020). Molecular recognition steps, changes in hydration of the ligand binding pocket and ligand dependent receptor configuration changes are also important considerations for D2 and D3 in particular when docking flexible ligands and establishing comparisons (Pal et al., 2019). Native system pharmacology studies are due to confirm the relevance of the observed *in vitro* differences. It would be indeed interesting to obtain a database of consistent functional information for all the ligands generated to further advance in the direction of new therapeutics. A re-evaluation of known DR ligands in the clinic on the basis of the latest available molecular model would be useful to help DR drug developers to build a more integrated view on the efforts, the tools and the information available and needed to move forward.

## Conclusion

This article reviews current knowledge on DR subtypes in SCZ, anatomical distribution, and new pharmacological tools that can help dissect out subtype-specific functions. The aspects of DR research described hereby are strictly related to SCZ or risk genes associated with it. What appears is that the current molecular understanding of Glutamate NMDA - DA interactions in SCZ has improved, but it is still insufficient in particular in brain areas like the ventral striatum and in relation to negative symptoms. A better understanding of the circuit(s) will possibly further reduce boundaries between cognitive and negative SCZ symptoms domains (Robison et al., 2020). The DA - NMDA research is also bringing the neurodevelopmental aspects of the SCZ disease to the core of current efforts and hopefully this will improve our understanding of SCZ disease onset and the relevance of DR research in SCZ animal models. It is therefore essential to integrate all the most recent DR findings and further discuss the NMDA Glutamate – DA dysregulation hypothesis for SCZ with a focus on the key interactors between the two systems (Kesby et al., 2018; Potkin et al., 2020). This may also help drug discovery to address the complexity of DR heterocomplexes in native systems using multiple intracellular markers and benefiting from the available more selective DR tools.

## Author Contributions

SG and JM contributed to the text, tables, and JM contributed the figure in the review manuscript.

## Conflict of Interest

The authors declare that the research was conducted in the absence of any commercial or financial relationships that could be construed as a potential conflict of interest.

## References

[B1] Aberg K. C. Kramer E. E. Schwartz S. (2020). Interplay between midbrain and dorsal anterior cingulate regions arbitrates lingering reward effects on memory encoding. Nat. Commun. 11 (1), 1829. 10.1038/s41467-020-15542-z 32286275 PMC7156375

[B2] Abi-Dargham A. (2020). From “bedside” to “bench” and back: A translational approach to studying dopamine dysfunction in schizophrenia. Neurosci. Biobehav. Rev. 110, 174–179. 10.1016/j.neubiorev.2018.12.003 30528375

[B3] Aguilar B. L. Forcelli P. A. Malkova L. (2018). Inhibition of the substantia nigra pars reticulata produces divergent effects on sensorimotor gating in rats and monkeys. Sci. Rep. 8 (1), 9369. 10.1038/s41598-018-27577-w 29921848 PMC6008324

[B4] Alvarez R. J. Pafundo D. E. Zold C. L. Belforte J. E. (2020). Interneuron NMDA Receptor Ablation Induces Hippocampus-Prefrontal Cortex Functional Hypoconnectivity after Adolescence in a Mouse Model of Schizophrenia. J. Neurosci. 40 (16), 3304–3317. 10.1523/JNEUROSCI.1897-19.2020 32205341 PMC7159887

[B5] Amato D. Kruyer A. Samaha A. N. Heinz A. (2019). Hypofunctional Dopamine Uptake and Antipsychotic Treatment-Resistant Schizophrenia. Front. Psychiatry 10, 314. 10.3389/fpsyt.2019.00314 31214054 PMC6557273

[B6] Anastasiades P. G. Boada C. Carter A. G. (2019). Cell-Type-Specific D1 Dopamine Receptor Modulation of Projection Neurons and Interneurons in the Prefrontal Cortex. Cereb. Cortex 29 (7), 3224–3242. 10.1093/cercor/bhy299 30566584 PMC6611468

[B7] Arango-Lievano M. Sensoy O. Borie A. Corbani M. Guillon G. Sokoloff P. (2016). A GIPC1-Palmitate Switch Modulates Dopamine Drd3 Receptor Trafficking and Signaling. Mol. Cell Biol. 36 (6), 1019–1031. 10.1128/MCB.00916-15 26787837 PMC4810479

[B8] Aringhieri S. Carli M. Kolachalam S. Verdesca V. Cini E. Rossi M. (2018). Molecular targets of atypical antipsychotics: From mechanism of action to clinical differences. Pharmacol. Ther. 192, 20–41. 10.1016/j.pharmthera.2018.06.012 29953902

[B9] Arnsten A. F. Girgis R. R. Gray D. L. Mailman R. B. (2017). Novel Dopamine Therapeutics for Cognitive Deficits in Schizophrenia. Biol. Psychiatry 81 (1), 67–77. 10.1016/j.biopsych.2015.12.028 26946382 PMC4949134

[B10] Artiges E. Leroy C. Dubol M. Prat M. Pepin A. Mabondo A. (2017). Striatal and Extrastriatal Dopamine Transporter Availability in Schizophrenia and Its Clinical Correlates: A Voxel-Based and High-Resolution PET Study. Schizophr. Bull. 43 (5), 1134–1142. 10.1093/schbul/sbw192 28177089 PMC5581903

[B11] Asif-Malik A. Hoener M. C. Canales J. J. (2017). Interaction Between the Trace Amine-Associated Receptor 1 and the Dopamine D2 Receptor Controls Cocaine’s Neurochemical Actions. Sci. Rep. 7 (1), 13901. 10.1038/s41598-017-14472-z 29066851 PMC5655641

[B12] Avram M. Brandl F. Cabello J. Leucht C. Scherr M. Mustafa M. (2019). Reduced striatal dopamine synthesis capacity in patients with schizophrenia during remission of positive symptoms. Brain 142 (6), 1813–1826. 10.1093/brain/awz093 31135051

[B13] Bailey M. R. Chun E. Schipani E. Balsam P. D. Simpson E. H. (2020). Dissociating the effects of dopamine D2 receptors on effort-based versus value-based decision making using a novel behavioral approach. Behav. Neurosci. 134 (2), 101–118. 10.1037/bne0000361 32175760 PMC7802819

[B14] Beaulieu J. M. Gainetdinov R. R. (2011). The physiology, signaling, and pharmacology of dopamine receptors. Pharmacol. Rev. 63 (1), 182–217. 10.1124/pr.110.002642 21303898

[B15] Berke J. D. (2018). What does dopamine mean? Nat. Neurosci. 21 (6), 787–793. 10.1038/s41593-018-0152-y 29760524 PMC6358212

[B16] Beutler L. R. Wanat M. J. Quintana A. Sanz E. Bamford N. S. Zweifel L. S. (2011). Balanced NMDA receptor activity in dopamine D1 receptor (D1R)- and D2R-expressing medium spiny neurons is required for amphetamine sensitization. Proc. Natl. Acad. Sci. U.S.A. 108 (10), 4206–4211. 10.1073/pnas.1101424108 21368124 PMC3054029

[B17] Blessing E. M. Murty V. P. Zeng B. Wang J. Davachi L. Goff D. C. (2020). Anterior Hippocampal-Cortical Functional Connectivity Distinguishes Antipsychotic Naive First-Episode Psychosis Patients From Controls and May Predict Response to Second-Generation Antipsychotic Treatment. Schizophr. Bull. 46 (3), 680–689. 10.1093/schbul/sbz076 31433843 PMC7147586

[B18] Bolton A. D. Constantine-Paton M. (2018). Synaptic Effects of Dopamine Breakdown and Their Relation to Schizophrenia-Linked Working Memory Deficits. Front. Synaptic Neurosci. 10, 16. 10.3389/fnsyn.2018.00016 29950984 PMC6008544

[B19] Bonifazi A. Yano H. Guerrero A. M. Kumar V. Hoffman A. F. Lupica C. R. (2019). Novel and Potent Dopamine D2 Receptor Go-Protein Biased Agonists. ACS Pharmacol. Transl. Sci. 2 (1), 52–65. 10.1021/acsptsci.8b00060 30775693 PMC6371206

[B20] Bontempi L. Savoia P. Bono F. Fiorentini C. Missale C. (2017). Dopamine D3 and acetylcholine nicotinic receptor heteromerization in midbrain dopamine neurons: Relevance for neuroplasticity. Eur. Neuropsychopharmacol. 27 (4), 313–324. 10.1016/j.euroneuro.2017.01.015 28187919

[B21] Borroto-Escuela D. O. Fuxe K. (2019). Oligomeric Receptor Complexes and Their Allosteric Receptor-Receptor Interactions in the Plasma Membrane Represent a New Biological Principle for Integration of Signals in the CNS. Front. Mol. Neurosci. 12, 230. 10.3389/fnmol.2019.00230 31607863 PMC6773811

[B22] Borroto-Escuela D. O. Pintsuk J. Schafer T. Friedland K. Ferraro L. Tanganelli S. (2016). Multiple D2 heteroreceptor complexes: new targets for treatment of schizophrenia. Ther. Adv. Psychopharmacol. 6 (2), 77–94. 10.1177/2045125316637570 27141290 PMC4837969

[B23] Borroto-Escuela D. O. Rodriguez D. Romero-Fernandez W. Kapla J. Jaiteh M. Ranganathan A. (2018). Mapping the Interface of a GPCR Dimer: A Structural Model of the A2A Adenosine and D2 Dopamine Receptor Heteromer. Front. Pharmacol. 9, 829. 10.3389/fphar.2018.00829 30214407 PMC6125358

[B24] Brady R. O. Jr. Gonsalvez I. Lee I. Ongur D. Seidman L. J. Schmahmann J. D. (2019). Cerebellar-Prefrontal Network Connectivity and Negative Symptoms in Schizophrenia. Am. J. Psychiatry 176 (7), 512–520. 10.1176/appi.ajp.2018.18040429 30696271 PMC6760327

[B25] Brignani S. Pasterkamp R. J. (2017). Neuronal Subset-Specific Migration and Axonal Wiring Mechanisms in the Developing Midbrain Dopamine System. Front. Neuroanat. 11, 55. 10.3389/fnana.2017.00055 28740464 PMC5502286

[B26] Brugger S. P. Angelescu I. Abi-Dargham A. Mizrahi R. Shahrezaei V. Howes O. D. (2020). Heterogeneity of Striatal Dopamine Function in Schizophrenia: Meta-analysis of Variance. Biol. Psychiatry 87 (3), 215–224. 10.1016/j.biopsych.2019.07.008 31561858

[B27] Bruns R. F. Mitchell S. N. Wafford K. A. Harper A. J. Shanks E. A. Carter G. (2018). Preclinical profile of a dopamine D1 potentiator suggests therapeutic utility in neurological and psychiatric disorders. Neuropharmacology 128, 351–365. 10.1016/j.neuropharm.2017.10.032 29102759

[B28] Bueschbell B. Barreto C. A. V. Preto A. J. Schiedel A. C. Moreira I. S. (2019). A Complete Assessment of Dopamine Receptor- Ligand Interactions through Computational Methods. Molecules 24 (7), 1196. 10.3390/molecules24071196 PMC6479630 30934701

[B29] Burke C. J. Soutschek A. Weber S. Raja Beharelle A. Fehr E. Haker H. (2018). Dopamine Receptor-Specific Contributions to the Computation of Value. Neuropsychopharmacology 43 (6), 1415–1424. 10.1038/npp.2017.302 29251282 PMC5916370

[B30] Butini S. Nikolic K. Kassel S. Bruckmann H. Filipic S. Agbaba D. (2016). Polypharmacology of dopamine receptor ligands. Prog. Neurobiol. 142, 68–103. 10.1016/j.pneurobio.2016.03.011 27234980

[B31] Cachia A. Cury C. Brunelin J. Plaze M. Delmaire C. Oppenheim C. (2020). Deviations in early hippocampus development contribute to visual hallucinations in schizophrenia. Transl. Psychiatry 10 (1), 102. 10.1038/s41398-020-0779-9 32214096 PMC7096500

[B32] Caravaggio F. Iwata Y. Kim J. Shah P. Gerretsen P. Remington G. (2020). What proportion of striatal D2 receptors are occupied by endogenous dopamine at baseline? A meta-analysis with implications for understanding antipsychotic occupancy. Neuropharmacology 163, 107591. 10.1016/j.neuropharm.2019.03.034 30940535

[B33] Carli M. Kolachalam S. Aringhieri S. Rossi M. Giovannini L. Maggio R. (2018). Dopamine D2 Receptors Dimers: How can we Pharmacologically Target Them? Curr. Neuropharmacol. 16 (2), 222–230. 10.2174/1570159X15666170518151127 28521704 PMC5883381

[B34] Castellani C. A. Melka M. G. Gui J. L. Gallo A. J. O’Reilly R. L. Singh S. M. (2017). Post-zygotic genomic changes in glutamate and dopamine pathway genes may explain discordance of monozygotic twins for schizophrenia. Clin. Transl. Med. 6 (1), 43. 10.1186/s40169-017-0174-1 29181591 PMC5704032

[B35] Castrellon J. J. Young J. S. Dang L. C. Cowan R. L. Zald D. H. Samanez-Larkin G. R. (2019). Mesolimbic dopamine D2 receptors and neural representations of subjective value. Sci. Rep. 9 (1), 20229. 10.1038/s41598-019-56858-1 31882947 PMC6934551

[B36] Chang W. H. Chen K. C. Tseng H. H. Chiu N. T. Lee I. H. Chen P. S. (2020). Bridging the associations between dopamine, brain volumetric variation and IQ in drug-naive schizophrenia. Schizophr. Res. 220, 248–253. 10.1016/j.schres.2020.03.005 32204972

[B37] Chen R. Jonas E. A. (2020). Dopamine fuels its own release. Nat. Neurosci. 23 (1), 1–2. 10.1038/s41593-019-0563-4 31844312

[B38] Chen X. McCorvy J. D. Fischer M. G. Butler K. V. Shen Y. Roth B. L. (2016). Discovery of G Protein-Biased D2 Dopamine Receptor Partial Agonists. J. Med. Chem. 59 (23), 10601–10618. 10.1021/acs.jmedchem.6b01208 27805392 PMC5148701

[B39] Chien E. Y. Liu W. Zhao Q. Katritch V. Han G. W. Hanson M. A. (2010). Structure of the human dopamine D3 receptor in complex with a D2/D3 selective antagonist. Science 330 (6007), 1091–1095. 10.1126/science.1197410 21097933 PMC3058422

[B40] Chuhma N. Mingote S. Kalmbach A. Yetnikoff L. Rayport S. (2017). Heterogeneity in Dopamine Neuron Synaptic Actions Across the Striatum and Its Relevance for Schizophrenia. Biol. Psychiatry 81 (1), 43–51. 10.1016/j.biopsych.2016.07.002 27692238 PMC5121049

[B41] Chung S. Lee E. J. Yun S. Choe H. K. Park S. B. Son H. J. (2014). Impact of circadian nuclear receptor REV-ERBalpha on midbrain dopamine production and mood regulation. Cell 157 (4), 858–868. 10.1016/j.cell.2014.03.039 24813609

[B42] Chung D. W. Chung Y. Bazmi H. H. Lewis D. A. (2018). Altered ErbB4 splicing and cortical parvalbumin interneuron dysfunction in schizophrenia and mood disorders. Neuropsychopharmacology 43 (12), 2478–2486. 10.1038/s41386-018-0169-7 30120408 PMC6180093

[B43] Cirnaru M. D. Melis C. Fanutza T. Naphade S. Tshilenge K. T. Muntean B. S. (2019). Nuclear Receptor Nr4a1 Regulates Striatal Striosome Development and Dopamine D1 Receptor Signaling. eNeuro 6 (5), 0305–19. 10.1523/ENEURO.0305-19.2019 PMC6787343 31541002

[B44] Clarkson R. L. Liptak A. T. Gee S. M. Sohal V. S. Bender K. J. (2017). D3 Receptors Regulate Excitability in a Unique Class of Prefrontal Pyramidal Cells. J. Neurosci. 37 (24), 5846–5860. 10.1523/JNEUROSCI.0310-17.2017 28522735 PMC5473204

[B45] Condon M. D. Platt N. J. Zhang Y. F. Roberts B. M. Clements M. A. Vietti-Michelina S. (2019). Plasticity in striatal dopamine release is governed by release-independent depression and the dopamine transporter. Nat. Commun. 10 (1), 4263. 10.1038/s41467-019-12264-9 31537790 PMC6753151

[B46] Correll C. U. Schooler N. R. (2020). Negative Symptoms in Schizophrenia: A Review and Clinical Guide for Recognition, Assessment, and Treatment. Neuropsychiatr. Dis. Treat. 16, 519–534. 10.2147/NDT.S225643 32110026 PMC7041437

[B47] Cortes A. Moreno E. Rodriguez-Ruiz M. Canela E. I. Casado V. (2016). Targeting the dopamine D3 receptor: an overview of drug design strategies. Expert Opin. Drug Discovery 11 (7), 641–664. 10.1080/17460441.2016.1185413 27135354

[B48] Coyle J. T. Balu D. Benneyworth M. Basu A. Roseman A. (2010). Beyond the dopamine receptor: novel therapeutic targets for treating schizophrenia. Dialogues Clin. Neurosci. 12 (3), 359–382. 20954431 10.31887/DCNS.2010.12.3/jcoyle PMC3181979

[B49] D’Ambrosio E. Jauhar S. Kim S. Veronese M. Rogdaki M. Pepper F. (2019). The relationship between grey matter volume and striatal dopamine function in psychosis: a multimodal (18)F-DOPA PET and voxel-based morphometry study. Mol. Psychiatry 10.1038/s41380-019-0570-6 PMC7610423 31690805

[B50] Daskalakis A. A. Zomorrodi R. Blumberger D. M. Rajji T. K. (2020). Evidence for prefrontal cortex hypofunctioning in schizophrenia through somatosensory evoked potentials. Schizophr. Res. 215, 197–203. 10.1016/j.schres.2019.10.030 31662233

[B51] David O. Barrera I. Gould N. Gal-Ben-Ari S. Rosenblum K. (2020). D1 Dopamine Receptor Activation Induces Neuronal eEF2 Pathway-Dependent Protein Synthesis. Front. Mol. Neurosci. 13, 67. 10.3389/fnmol.2020.00067 32499677 PMC7242790

[B52] De Vries L. Finana F. Cathala C. Ronsin B. Cussac D. (2019). Innovative Bioluminescence Resonance Energy Transfer Assay Reveals Differential Agonist-Induced D2 Receptor Intracellular Trafficking and Arrestin-3 Recruitment. Mol. Pharmacol. 96 (3), 308–319. 10.1124/mol.119.115998 31266815

[B53] Delevich K. Jaaro-Peled H. Penzo M. Sawa A. Li B. (2020). Parvalbumin Interneuron Dysfunction in a Thalamo-Prefrontal Cortical Circuit in Disc1 Locus Impairment Mice. eNeuro 7 (2). 10.1523/ENEURO.0496-19.2020 PMC7054897 32029441

[B54] Desai S. J. Allman B. L. Rajakumar N. (2017). Combination of behaviorally sub-effective doses of glutamate NMDA and dopamine D1 receptor antagonists impairs executive function. Behav. Brain Res. 323, 24–31. 10.1016/j.bbr.2017.01.030 28115219

[B55] Dreyer J. K. Herrik K. F. Berg R. W. Hounsgaard J. D. (2010). Influence of phasic and tonic dopamine release on receptor activation. J. Neurosci. 30 (42), 14273–14283. 10.1523/JNEUROSCI.1894-10.2010 20962248 PMC6634758

[B56] Ebersole B. Petko J. Woll M. Murakami S. Sokolina K. Wong V. (2015). Effect of C-Terminal S-Palmitoylation on D2 Dopamine Receptor Trafficking and Stability. PloS One 10 (11), e0140661. 10.1371/journal.pone.0140661 26535572 PMC4633242

[B57] El Mestikawy S. Wallen-Mackenzie A. Fortin G. M. Descarries L. Trudeau L. E. (2011). From glutamate co-release to vesicular synergy: vesicular glutamate transporters. Nat. Rev. Neurosci. 12 (4), 204–216. 10.1038/nrn2969 21415847

[B58] Enge S. Sach M. Reif A. Lesch K. P. Miller R. Fleischhauer M. (2020). Cumulative Dopamine Genetic Score predicts behavioral and electrophysiological correlates of response inhibition via interactions with task demand. Cognit. Affect. Behav. Neurosci. 20 (1), 59–75. 10.3758/s13415-019-00752-w 31802408 PMC7012812

[B59] Eshel N. Bukwich M. Rao V. Hemmelder V. Tian J. Uchida N. (2015). Arithmetic and local circuitry underlying dopamine prediction errors. Nature 525 (7568), 243–246. 10.1038/nature14855 26322583 PMC4567485

[B60] Fan L. Tan L. Chen Z. Qi J. Nie F. Luo Z. (2020). Haloperidol bound D2 dopamine receptor structure inspired the discovery of subtype selective ligands. Nat. Commun. 11 (1), 1074. 10.1038/s41467-020-14884-y 32103023 PMC7044277

[B61] Fareri D. S. Gabard-Durnam L. Goff B. Flannery J. Gee D. G. Lumian D. S. (2017). Altered ventral striatal-medial prefrontal cortex resting-state connectivity mediates adolescent social problems after early institutional care. Dev. Psychopathol. 29 (5), 1865–1876. 10.1017/S0954579417001456 29162189 PMC5957481

[B62] Faron-Gorecka A. Kusmider M. Solich J. Gorecki A. Dziedzicka-Wasylewska M. (2020). Genetic variants in dopamine receptors influence on heterodimerization in the context of antipsychotic drug action. Prog. Mol. Biol. Transl. Sci. 169, 279–296. 10.1016/bs.pmbts.2019.11.008 31952689

[B63] Ferraro M. Decherchi S. De Simone A. Recanatini M. Cavalli A. Bottegoni G. (2020). Multi-target dopamine D3 receptor modulators: Actionable knowledge for drug design from molecular dynamics and machine learning. Eur. J. Med. Chem. 188, 111975. 10.1016/j.ejmech.2019.111975 31940507

[B64] Ferre S. Bonaventura J. Zhu W. Hatcher-Solis C. Taura J. Quiroz C. (2018). Essential Control of the Function of the Striatopallidal Neuron by Pre-coupled Complexes of Adenosine A2A-Dopamine D2 Receptor Heterotetramers and Adenylyl Cyclase. Front. Pharmacol. 9, 243. 10.3389/fphar.2018.00243 29686613 PMC5900444

[B65] Foster D. J. Conn P. J. (2017). Allosteric Modulation of GPCRs: New Insights and Potential Utility for Treatment of Schizophrenia and Other CNS Disorders. Neuron 94 (3), 431–446. 10.1016/j.neuron.2017.03.016 28472649 PMC5482176

[B66] Frankle W. G. Narendran R. (2020). Distinguishing Schizophrenia Subtypes: Can Dopamine Imaging Improve the Signal-to-Noise Ratio? Biol. Psychiatry 87 (3), 197–199. 10.1016/j.biopsych.2019.11.004 31908286

[B67] Fusar-Poli P. Meyer-Lindenberg A. (2013). Striatal presynaptic dopamine in schizophrenia, Part I: meta-analysis of dopamine active transporter (DAT) density. Schizophr. Bull. 39 (1), 22–32. 10.1093/schbul/sbr111 22282456 PMC3523907

[B68] Gienger M. Hubner H. Lober S. Konig B. Gmeiner P. (2020). Structure-based development of caged dopamine D2/D3 receptor antagonists. Sci. Rep. 10 (1), 829. 10.1038/s41598-020-57770-9 31965029 PMC6972920

[B69] Girgenti M. J. LoTurco J. J. Maher B. J. (2012). ZNF804a regulates expression of the schizophrenia-associated genes PRSS16, COMT, PDE4B, and DRD2. PloS One 7 (2), e32404. 10.1371/journal.pone.0032404 22384243 PMC3288100

[B70] Girgis R. R. Forbes A. Abi-Dargham A. Slifstein M. (2020). A positron emission tomography occupancy study of brexpiprazole at dopamine D2 and D3 and serotonin 5-HT1A and 5-HT2A receptors, and serotonin reuptake transporters in subjects with schizophrenia. Neuropsychopharmacology 45 (5), 786–792. 10.1038/s41386-019-0590-6 31847007 PMC7075883

[B71] Gonzalez-Burgos G. Miyamae T. Krimer Y. Gulchina Y. Pafundo D. E. Krimer O. (2019). Distinct Properties of Layer 3 Pyramidal Neurons from Prefrontal and Parietal Areas of the Monkey Neocortex. J. Neurosci. 39 (37), 7277–7290. 10.1523/JNEUROSCI.1210-19.2019 31341029 PMC6759021

[B72] Grace A. A. Gomes F. V. (2019). The Circuitry of Dopamine System Regulation and its Disruption in Schizophrenia: Insights Into Treatment and Prevention. Schizophr. Bull. 45 (1), 148–157. 10.1093/schbul/sbx199 29385549 PMC6293217

[B73] Grace A. A. (2016). Dysregulation of the dopamine system in the pathophysiology of schizophrenia and depression. Nat. Rev. Neurosci. 17 (8), 524–532. 10.1038/nrn.2016.57 27256556 PMC5166560

[B74] Gray D. L. Allen J. A. Mente S. O’Connor R. E. DeMarco G. J. Efremov I. (2018). Impaired beta-arrestin recruitment and reduced desensitization by non-catechol agonists of the D1 dopamine receptor. Nat. Commun. 9 (1), 674. 10.1038/s41467-017-02776-7 29445200 PMC5813016

[B75] Grea H. Bouchet D. Rogemond V. Hamdani N. Le Guen E. Tamouza R. (2019). Human Autoantibodies Against N-Methyl-D-Aspartate Receptor Modestly Alter Dopamine D1 Receptor Surface Dynamics. Front. Psychiatry 10, 670. 10.3389/fpsyt.2019.00670 31572244 PMC6754069

[B76] Gross J. D. Kaski S. W. Schroer A. B. Wix K. A. Siderovski D. P. Setola V. (2018). Regulator of G protein signaling-12 modulates the dopamine transporter in ventral striatum and locomotor responses to psychostimulants. J. Psychopharmacol. 32 (2), 191–203. 10.1177/0269881117742100 29364035 PMC5942192

[B77] Guitart X. Moreno E. Rea W. Sanchez-Soto M. Cai N. S. Quiroz C. (2019). Biased G Protein-Independent Signaling of Dopamine D1-D3 Receptor Heteromers in the Nucleus Accumbens. Mol. Neurobiol. 56 (10), 6756–6769. 10.1007/s12035-019-1564-8 30919214 PMC6728209

[B78] Guma E. Rocchetti J. Devenyi G. A. Tanti A. Mathieu A. P. Lerch J. P. (2019). Role of D3 dopamine receptors in modulating neuroanatomical changes in response to antipsychotic administration. Sci. Rep. 9 (1), 7850. 10.1038/s41598-019-43955-4 31127135 PMC6534671

[B79] Gutierrez-Arenas O. Eriksson O. Kotaleski J. H. (2014). Segregation and crosstalk of D1 receptor-mediated activation of ERK in striatal medium spiny neurons upon acute administration of psychostimulants. PloS Comput. Biol. 10 (1), e1003445. 10.1371/journal.pcbi.1003445 24499932 PMC3907292

[B80] Hall A. Provins L. Valade A. (2019). Novel Strategies To Activate the Dopamine D1 Receptor: Recent Advances in Orthosteric Agonism and Positive Allosteric Modulation. J. Med. Chem. 62 (1), 128–140. 10.1021/acs.jmedchem.8b01767 30525590

[B81] Hamid A. A. Pettibone J. R. Mabrouk O. S. Hetrick V. L. Schmidt R. Vander Weele C. M. (2016). Mesolimbic dopamine signals the value of work. Nat. Neurosci. 19 (1), 117–126. 10.1038/nn.4173 26595651 PMC4696912

[B82] Han S. Becker B. Duan X. Cui Q. Xin F. Zong X. (2020). Distinct striatum pathways connected to salience network predict symptoms improvement and resilient functioning in schizophrenia following risperidone monotherapy. Schizophr. Res. 215, 89–96. 10.1016/j.schres.2019.11.017 31759811

[B83] Harada A. Kaushal N. Suzuki K. Nakatani A. Bobkov K. Vekich J. A. (2020). Balanced Activation of Striatal Output Pathways by Faster Off-Rate PDE10A Inhibitors Elicits Not Only Antipsychotic-Like Effects But Also Procognitive Effects in Rodents. Int. J. Neuropsychopharmacol. 23 (2), 96–107. 10.1093/ijnp/pyz056 31689714 PMC7098246

[B84] He D. Lasek A. W. (2020). Anaplastic Lymphoma Kinase Regulates Internalization of the Dopamine D2 Receptor. Mol. Pharmacol. 97 (2), 123–131. 10.1124/mol.119.117473 31734646 PMC6964149

[B85] Hikima T. Garcia-Munoz M. Arbuthnott G. W. (2016). Presynaptic D1 heteroreceptors and mGlu autoreceptors act at individual cortical release sites to modify glutamate release. Brain Res. 1639, 74–87. 10.1016/j.brainres.2016.02.042 26944299

[B86] Hu J. L. Liu G. Li Y. C. Gao W. J. Huang Y. Q. (2010). Dopamine D1 receptor-mediated NMDA receptor insertion depends on Fyn but not Src kinase pathway in prefrontal cortical neurons. Mol. Brain 3, 20. 10.1186/1756-6606-3-20 20569495 PMC2902469

[B87] Huang R. Griffin S. A. Taylor M. Vangveravong S. Mach R. H. Dillon G. H. (2013). The effect of SV 293, a D2 dopamine receptor-selective antagonist, on D2 receptor-mediated GIRK channel activation and adenylyl cyclase inhibition. Pharmacology 92 (1-2), 84–89. 10.1159/000351971 23942137 PMC12013381

[B88] Huang Y. C. Lee Y. Lee C. Y. Lin P. Y. Hung C. F. Lee S. Y. (2020). Defining cognitive and functional profiles in schizophrenia and affective disorders. BMC Psychiatry 20 (1), 39. 10.1186/s12888-020-2459-y 32005199 PMC6995055

[B89] Hubner H. Schellhorn T. Gienger M. Schaab C. Kaindl J. Leeb L. (2016). Structure-guided development of heterodimer-selective GPCR ligands. Nat. Commun. 7, 12298. 10.1038/ncomms12298 27457610 PMC4963535

[B90] Hunger L. Kumar A. Schmidt R. (2020). Abundance Compensates Kinetics: Similar Effect of Dopamine Signals on D1 and D2 Receptor Populations. J. Neurosci. 40 (14), 2868–2881. 10.1523/JNEUROSCI.1951-19.2019 32071139 PMC7117896

[B91] Hunnicutt B. J. Jongbloets B. C. Birdsong W. T. Gertz K. J. Zhong H. Mao T. (2016). A comprehensive excitatory input map of the striatum reveals novel functional organization. Elife 5. 10.7554/eLife.19103 PMC5207773 27892854

[B92] Hwang R. Tiwari A. K. Zai C. C. Felsky D. Remington E. Wallace T. (2012). Dopamine D4 and D5 receptor gene variant effects on clozapine response in schizophrenia: replication and exploration. Prog. Neuropsychopharmacol. Biol. Psychiatry 37 (1), 62–75. 10.1016/j.pnpbp.2011.11.018 22203087

[B93] Iino Y. Sawada T. Yamaguchi K. Tajiri M. Ishii S. Kasai H. (2020). Dopamine D2 receptors in discrimination learning and spine enlargement. Nature 579 (7800), 555–560. 10.1038/s41586-020-2115-1 32214250

[B94] Jackson M. F. (2020). Epigenetic Mechanism Links NMDA Receptor Hypofunction and Cognitive Deficits in Schizophrenia to D2 Receptors. Biol. Psychiatry 87 (8), 692–694. 10.1016/j.biopsych.2020.01.024 32216901

[B95] Jacobi A. A. Halawani S. Lynch D. R. Lin H. (2019). Neuronal serine racemase associates with Disrupted-In-Schizophrenia-1 and DISC1 agglomerates: Implications for schizophrenia. Neurosci. Lett. 692, 107–114. 10.1016/j.neulet.2018.10.055 30391323 PMC6402792

[B96] Jezequel J. Johansson E. M. Dupuis J. P. Rogemond V. Grea H. Kellermayer B. (2017). Dynamic disorganization of synaptic NMDA receptors triggered by autoantibodies from psychotic patients. Nat. Commun. 8 (1), 1791. 10.1016/j.tins.2018.05.002 29176681 PMC5702610

[B97] Jezequel J. Johansson E. M. Leboyer M. Groc L. (2018). Pathogenicity of Antibodies against NMDA Receptor: Molecular Insights into Autoimmune Psychosis. Trends Neurosci. 41 (8), 502–511. 10.1016/j.tins.2018.05.002 29807730

[B98] Jiang S. Z. Xu W. Emery A. C. Gerfen C. R. Eiden M. V. Eiden L. E. (2017). NCS-Rapgef2, the Protein Product of the Neuronal Rapgef2 Gene, Is a Specific Activator of D1 Dopamine Receptor-Dependent ERK Phosphorylation in Mouse Brain. eNeuro 4 (5). 10.1523/ENEURO.0248-17.2017 PMC5611689 28948210

[B99] Kaczor A. A. Jorg M. Capuano B. (2016). The dopamine D2 receptor dimer and its interaction with homobivalent antagonists: homology modeling, docking and molecular dynamics. J. Mol. Model. 22 (9), 203. 10.1007/s00894-016-3065-2 27491852 PMC5023759

[B100] Karadurmus D. Rial D. De Backer J. F. Communi D. de Kerchove d’Exaerde A. Schiffmann S. N. (2019). GPRIN3 Controls Neuronal Excitability, Morphology, and Striatal-Dependent Behaviors in the Indirect Pathway of the Striatum. J. Neurosci. 39 (38), 7513–7528. 10.1523/JNEUROSCI.2454-18.2019 31363062 PMC6750934

[B101] Kasai R. S. Ito S. V. Awane R. M. Fujiwara T. K. Kusumi A. (2018). The Class-A GPCR Dopamine D2 Receptor Forms Transient Dimers Stabilized by Agonists: Detection by Single-Molecule Tracking. Cell Biochem. Biophys. 76 (1-2), 29–37. 10.1007/s12013-017-0829-y 29116599 PMC5913388

[B102] Kern A. Mavrikaki M. Ullrich C. Albarran-Zeckler R. Brantley A. F. Smith R. G. (2015). Hippocampal Dopamine/DRD1 Signaling Dependent on the Ghrelin Receptor. Cell 163 (5), 1176–1190. 10.1016/j.cell.2015.10.062 26590421 PMC4937825

[B103] Kesby J. P. Eyles D. W. McGrath J. J. Scott J. G. (2018). Dopamine, psychosis and schizophrenia: the widening gap between basic and clinical neuroscience. Transl. Psychiatry 8 (1), 30. 10.1038/s41398-017-0071-9 29382821 PMC5802623

[B104] Khlghatyan J. Quintana C. Parent M. Beaulieu J. M. (2019). High Sensitivity Mapping of Cortical Dopamine D2 Receptor Expressing Neurons. Cereb. Cortex 29 (9), 3813–3827. 10.1093/cercor/bhy261 30295716 PMC6686758

[B105] Kim S. Jung W. H. Howes O. D. Veronese M. Turkheimer F. E. Lee Y. S. (2019). Frontostriatal functional connectivity and striatal dopamine synthesis capacity in schizophrenia in terms of antipsychotic responsiveness: an [(18)F]DOPA PET and fMRI study. Psychol. Med. 49 (15), 2533–2542. 10.1017/S0033291718003471 30460891

[B106] Koblan K. S. Kent J. Hopkins S. C. Krystal J. H. Cheng H. Goldman R. (2020). A Non-D2-Receptor-Binding Drug for the Treatment of Schizophrenia. N Engl. J. Med. 382 (16), 1497–1506. 10.1056/NEJMoa1911772 32294346

[B107] Kontaris I. East B. S. Wilson D. A. (2020). Behavioral and Neurobiological Convergence of Odor, Mood and Emotion: A Review. Front. Behav. Neurosci. 14, 35. 10.3389/fnbeh.2020.00035 32210776 PMC7076187

[B108] Kos M. Z. Duan J. Sanders A. R. Blondell L. Drigalenko E. I. Carless M. A. (2018). Dopamine perturbation of gene co-expression networks reveals differential response in schizophrenia for translational machinery. Transl. Psychiatry 8 (1), 278. 10.1038/s41398-018-0325-1 30546022 PMC6293320

[B109] Kotarska A. Fernandes L. Kleene R. Schachner M. (2020). Cell adhesion molecule close homolog of L1 binds to the dopamine receptor D2 and inhibits the internalization of its short isoform. FASEB J. 34 (4), 4832–4851. 10.1096/fj.201900577RRRR 32052901

[B110] Ladepeche L. Dupuis J. P. Bouchet D. Doudnikoff E. Yang L. Campagne Y. (2013). Single-molecule imaging of the functional crosstalk between surface NMDA and dopamine D1 receptors. Proc. Natl. Acad. Sci. U.S.A. 110 (44), 18005–18010. 10.1073/pnas.1310145110 24127604 PMC3816474

[B111] Lak A. Okun M. Moss M. M. Gurnani H. Farrell K. Wells M. J. (2020). Dopaminergic and Prefrontal Basis of Learning from Sensory Confidence and Reward Value. Neuron 105 (4), 700–711. 10.1016/j.neuron.2019.11.018 31859030 PMC7031700

[B112] Lane J. R. Abramyan A. M. Adhikari P. Keen A. C. Lee K. H. Sanchez J. (2020). Distinct inactive conformations of the dopamine D2 and D3 receptors correspond to different extents of inverse agonism. Elife 9. 10.7554/eLife.52189 PMC7053997 31985399

[B113] Lebowitz J. J. Khoshbouei H. (2020). Heterogeneity of dopamine release sites in health and degeneration. Neurobiol. Dis. 134, 104633. 10.1016/j.nbd.2019.104633 31698055 PMC6980674

[B114] Leggio G. M. Torrisi S. A. Mastrogiacomo R. Mauro D. Chisari M. Devroye C. (2019). The epistatic interaction between the dopamine D3 receptor and dysbindin-1 modulates higher-order cognitive functions in mice and humans. Mol. Psychiatry. 10.1038/s41380-019-0511-4 31492942

[B115] Leo D. Mus L. Espinoza S. Hoener M. C. Sotnikova T. D. Gainetdinov R. R. (2014). Taar1-mediated modulation of presynaptic dopaminergic neurotransmission: role of D2 dopamine autoreceptors. Neuropharmacology 81, 283–291. 10.1016/j.neuropharm.2014.02.007 24565640

[B116] Li A. Zalesky A. Yue W. Howes O. Yan H. Liu Y. (2020). A neuroimaging biomarker for striatal dysfunction in schizophrenia. Nat. Med. 26 (4), 558–565. 10.1038/s41591-020-0793-8 32251404

[B117] Liu X. Y. Mao L. M. Zhang G. C. Papasian C. J. Fibuch E. E. Lan H. X. (2009). Activity-dependent modulation of limbic dopamine D3 receptors by CaMKII. Neuron 61 (3), 425–438. 10.1016/j.neuron.2008.12.015 19217379 PMC2650276

[B118] Lohani S. Martig A. K. Underhill S. M. DeFrancesco A. Roberts M. J. Rinaman L. (2018). Burst activation of dopamine neurons produces prolonged post-burst availability of actively released dopamine. Neuropsychopharmacology 43 (10), 2083–2092. 10.1038/s41386-018-0088-7 29795245 PMC6098082

[B119] Lohani S. Martig A. K. Deisseroth K. Witten I. B. Moghaddam B. (2019). Dopamine Modulation of Prefrontal Cortex Activity Is Manifold and Operates at Multiple Temporal and Spatial Scales. Cell Rep. 27 (1), 99–114 e6. 10.1016/j.celrep.2019.03.012 30943418 PMC11884507

[B120] Lucarelli M. Di Pietro C. La Sala G. Fiorenza M. T. Marazziti D. Canterini S. (2019). Anomalies in Dopamine Transporter Expression and Primary Cilium Distribution in the Dorsal Striatum of a Mouse Model of Niemann-Pick C1 Disease. Front. Cell Neurosci. 13, 226. 10.3389/fncel.2019.00226 31178699 PMC6544041

[B121] Lukasiewicz S. Blasiak E. Szafran-Pilch K. Dziedzicka-Wasylewska M. (2016). Dopamine D2 and serotonin 5-HT1A receptor interaction in the context of the effects of antipsychotics - in vitro studies. J. Neurochem. 137 (4), 549–560. 10.1111/jnc.13582 26876117

[B122] Luo Y. J. Li Y. D. Wang L. Yang S. R. Yuan X. S. Wang J. (2018). Nucleus accumbens controls wakefulness by a subpopulation of neurons expressing dopamine D1 receptors. Nat. Commun. 9 (1), 1576. 10.1038/s41467-018-03889-3 29679009 PMC5910424

[B123] Ma J. Zhang H. Zhang X. Lei M. (2019). 3D-QSAR studies of D3R antagonists and 5-HT1AR agonists. J. Mol. Graph Model. 86, 132–141. 10.1016/j.jmgm.2018.10.013 30359859

[B124] Madras B. K. (2013). History of the discovery of the antipsychotic dopamine D2 receptor: a basis for the dopamine hypothesis of schizophrenia. J. Hist. Neurosci. 22 (1), 62–78. 10.1080/0964704X.2012.678199 23323533

[B125] Marcott P. F. Mamaligas A. A. Ford C. P. (2014). Phasic dopamine release drives rapid activation of striatal D2-receptors. Neuron 84 (1), 164–176. 10.1016/j.neuron.2014.08.058 25242218 PMC4325987

[B126] Marcott P. F. Gong S. Donthamsetti P. Grinnell S. G. Nelson M. N. Newman A. H. (2018). Regional Heterogeneity of D2-Receptor Signaling in the Dorsal Striatum and Nucleus Accumbens. Neuron 98 (3), 575–87 e4. 10.1016/j.neuron.2018.03.038 29656874 PMC6048973

[B127] Matera C. Bono F. Pelucchi S. Collo G. Bontempi L. Gotti C. (2019). The novel hybrid agonist HyNDA-1 targets the D3R-nAChR heteromeric complex in dopaminergic neurons. Biochem. Pharmacol. 163, 154–168. 10.1016/j.bcp.2019.02.019 30772268

[B128] McCutcheon R. A. Abi-Dargham A. Howes O. D. (2019). Schizophrenia, Dopamine and the Striatum: From Biology to Symptoms. Trends Neurosci. 42 (3), 205–220. 10.1016/j.tins.2018.12.004 30621912 PMC6401206

[B129] McCutcheon R. A. Krystal J. H. Howes O. D. (2020). Dopamine and glutamate in schizophrenia: biology, symptoms and treatment. World Psychiatry 19 (1), 15–33. 10.1002/wps.20693 31922684 PMC6953551

[B130] Meier M. A. Lemercier C. E. Kulisch C. Kiss B. Lendvai B. Adham N. (2020). The novel antipsychotic cariprazine stabilizes gamma oscillations in rat hippocampal slices. Br. J. Pharmacol. 177 (7), 1622–1634. 10.1111/bph.14923 31722437 PMC7060372

[B131] Min C. Zheng M. Zhang X. Guo S. Kwon K. J. Shin C. Y. (2015). N-linked Glycosylation on the N-terminus of the dopamine D2 and D3 receptors determines receptor association with specific microdomains in the plasma membrane. Biochim. Biophys. Acta 1853 (1), 41–51. 10.1016/j.bbamcr.2014.09.024 25289757

[B132] Mitelman S. A. Buchsbaum M. S. Christian B. T. Merrill B. M. Buchsbaum B. R. Mukherjee J. (2018). Dopamine receptor density and white mater integrity: (18)F-fallypride positron emission tomography and diffusion tensor imaging study in healthy and schizophrenia subjects. Brain Imaging Behav. 10.1007/s11682-018-0012-0 30523488

[B133] Mohebi A. Pettibone J. R. Hamid A. A. Wong J. T. Vinson L. T. Patriarchi T. (2019). Dissociable dopamine dynamics for learning and motivation. Nature 570 (7759), 65–70. 10.1038/s41586-019-1235-y 31118513 PMC6555489

[B134] Molinaro L. Hui P. Tan M. Mishra R. K. (2015). Role of presynaptic phosphoprotein synapsin II in schizophrenia. World J. Psychiatry 5 (3), 260–272. 10.5498/wjp.v5.i3.260 26425441 PMC4582303

[B135] Moritz A. E. Free R. B. Sibley D. R. (2018). Advances and challenges in the search for D2 and D3 dopamine receptor-selective compounds. Cell Signal. 41, 75–81. 10.1016/j.cellsig.2017.07.003 28716664 PMC5722689

[B136] Nai Q. Li S. Wang S. H. Liu J. Lee F. J. Frankland P. W. (2010). Uncoupling the D1-N-methyl-D-aspartate (NMDA) receptor complex promotes NMDA-dependent long-term potentiation and working memory. Biol. Psychiatry 67 (3), 246–254. 10.1016/j.biopsych.2009.08.011 19846062

[B137] Nair A. G. Gutierrez-Arenas O. Eriksson O. Jauhiainen A. Blackwell K. T. Kotaleski J. H. (2014). Modeling intracellular signaling underlying striatal function in health and disease. Prog. Mol. Biol. Transl. Sci. 123, 277–304. 10.1016/B978-0-12-397897-4.00013-9 24560149 PMC4120139

[B138] Nair A. G. Castro L. R. V. El Khoury M. Gorgievski V. Giros B. Tzavara E. T. (2019). The high efficacy of muscarinic M4 receptor in D1 medium spiny neurons reverses striatal hyperdopaminergia. Neuropharmacology 146, 74–83. 10.1016/j.neuropharm.2018.11.029 30468798

[B139] Nakao K. Jeevakumar V. Jiang S. Z. Fujita Y. Diaz N. B. Pretell Annan C. A. (2019). Schizophrenia-Like Dopamine Release Abnormalities in a Mouse Model of NMDA Receptor Hypofunction. Schizophr. Bull. 45 (1), 138–147. 10.1093/schbul/sby003 29394409 PMC6293233

[B140] Nicoletti F. Orlando R. Di Menna L. Cannella M. Notartomaso S. Mascio G. (2019). Targeting mGlu Receptors for Optimization of Antipsychotic Activity and Disease-Modifying Effect in Schizophrenia. Front. Psychiatry 10, 49. 10.3389/fpsyt.2019.00049 30890967 PMC6413697

[B141] Nishi A. Kuroiwa M. Shuto T. (2011). Mechanisms for the modulation of dopamine d(1) receptor signaling in striatal neurons. Front. Neuroanat. 5, 43. 10.3389/fnana.2011.00043 21811441 PMC3140648

[B142] Ohira K. (2020). Dopamine as a growth differentiation factor in the mammalian brain. Neural Regener. Res. 15 (3), 390–393. 10.4103/1673-5374.266052 PMC6921355 31571646

[B143] Oishi K. Niitsu T. Kanahara N. Hashimoto T. Komatsu H. Sasaki T. (2020). Genetic combination risk for schizophrenia. Schizophr. Res. 215, 473–474. 10.1016/j.schres.2019.08.021 31477374

[B144] Onishi T. Sakamoto H. Namiki S. Hirose K. (2018). The Altered Supramolecular Structure of Dopamine D2 Receptors in Disc1-deficient Mice. Sci. Rep. 8 (1), 1692. 10.1038/s41598-018-20090-0 29374282 PMC5785963

[B145] Pal R. K. Gadhiya S. Ramsey S. Cordone P. Wickstrom L. Harding W. W. (2019). Inclusion of enclosed hydration effects in the binding free energy estimation of dopamine D3 receptor complexes. PloS One 14 (9), e0222902. 10.1371/journal.pone.0222902 31568493 PMC6768453

[B146] Papenberg G. Karalija N. Salami A. Rieckmann A. Andersson M. Axelsson J. (2020). Balance between Transmitter Availability and Dopamine D2 Receptors in Prefrontal Cortex Influences Memory Functioning. Cereb. Cortex 30 (3), 989–1000. 10.1093/cercor/bhz142 31504282

[B147] Park S. M. Chen M. Schmerberg C. M. Dulman R. S. Rodriguiz R. M. Caron M. G. (2016). Effects of beta-Arrestin-Biased Dopamine D2 Receptor Ligands on Schizophrenia-Like Behavior in Hypoglutamatergic Mice. Neuropsychopharmacology 41 (3), 704–715. 10.1038/npp.2015.196 26129680 PMC4707817

[B148] Park J. H. Hong J. S. Kim S. M. Min K. J. Chung U. S. Han D. H. (2019). Effects of Amisulpride Adjunctive Therapy on Working Memory and Brain Metabolism in the Frontal Cortex of Patients with Schizophrenia: A Preliminary Positron Emission Tomography/Computerized Tomography Investigation. Clin. Psychopharmacol. Neurosci. 17 (2), 250–260. 10.9758/cpn.2019.17.2.250 30905125 PMC6478094

[B149] Pei Y. Asif-Malik A. Canales J. J. (2016). Trace Amines and the Trace Amine-Associated Receptor 1: Pharmacology, Neurochemistry, and Clinical Implications. Front. Neurosci. 10, 148. 10.3389/fnins.2016.00148 27092049 PMC4820462

[B150] Periclou A. Willavize S. Jaworowicz D. Passarell J. Carrothers T. Ghahramani P. (2020). Relationship Between Plasma Concentrations and Clinical Effects of Cariprazine in Patients With Schizophrenia or Bipolar Mania. Clin. Transl. Sci. 13 (2), 362–371. 10.1111/cts.12720 31664765 PMC7070889

[B151] Perreault M. L. Hasbi A. O’Dowd B. F. George S. R. (2011). The dopamine d1-d2 receptor heteromer in striatal medium spiny neurons: evidence for a third distinct neuronal pathway in Basal Ganglia. Front. Neuroanat. 5, 31. 10.3389/fnana.2011.00031 21747759 PMC3130461

[B152] Petralia M. C. Ciurleo R. Saraceno A. Pennisi M. Basile M. S. Fagone P. (2020). Meta-Analysis of Transcriptomic Data of Dorsolateral Prefrontal Cortex and of Peripheral Blood Mononuclear Cells Identifies Altered Pathways in Schizophrenia. Genes (Basel) 11 (4), 390. 10.3390/genes11040390 PMC7230488 32260267

[B153] Petty A. Cui X. Tesiram Y. Kirik D. Howes O. Eyles D. (2019). Enhanced Dopamine in Prodromal Schizophrenia (EDiPS): a new animal model of relevance to schizophrenia. NPJ Schizophr. 5 (1), 6. 10.1038/s41537-019-0074-z 30926827 PMC6441087

[B154] Potkin S. G. Kane J. M. Correll C. U. Lindenmayer J. P. Agid O. Marder S. R. (2020). The neurobiology of treatment-resistant schizophrenia: paths to antipsychotic resistance and a roadmap for future research. NPJ Schizophr. 6 (1), 1. 10.1038/s41537-019-0090-z 31911624 PMC6946650

[B155] Provenzano F. A. Guo J. Wall M. M. Feng X. Sigmon H. C. Brucato G. (2020). Hippocampal Pathology in Clinical High-Risk Patients and the Onset of Schizophrenia. Biol. Psychiatry 87 (3), 234–242. 10.1016/j.biopsych.2019.09.022 31771861

[B156] Pulido D. Casado-Anguera V. Perez-Benito L. Moreno E. Cordomi A. Lopez L. (2018). Design of a True Bivalent Ligand with Picomolar Binding Affinity for a G Protein-Coupled Receptor Homodimer. J. Med. Chem. 61 (20), 9335–9346. 10.1021/acs.jmedchem.8b01249 30257092 PMC9366271

[B157] Purves-Tyson T. D. Owens S. J. Rothmond D. A. Halliday G. M. Double K. L. Stevens J. (2017). Putative presynaptic dopamine dysregulation in schizophrenia is supported by molecular evidence from post-mortem human midbrain. Transl. Psychiatry 7 (1), e1003. 10.1038/tp.2016.257 28094812 PMC5545725

[B158] Qian M. Wouters E. Dalton J. A. R. Risseeuw M. D. P. Crans R. A. J. Stove C. (2018a). Synthesis toward Bivalent Ligands for the Dopamine D2 and Metabotropic Glutamate 5 Receptors. J. Med. Chem. 61 (18), 8212–8225. 10.1021/acs.jmedchem.8b00671 30180563

[B159] Qian M. Vasudevan L. Huysentruyt J. Risseeuw M. D. P. Stove C. Vanderheyden P. M. L. (2018b). Design, Synthesis, and Biological Evaluation of Bivalent Ligands Targeting Dopamine D2 -Like Receptors and the mu-Opioid Receptor. ChemMedChem 13 (9), 944–956. 10.1002/cmdc.201700787 29451744

[B160] Quintana C. Beaulieu J. M. (2019). A fresh look at cortical dopamine D2 receptor expressing neurons. Pharmacol. Res. 139, 440–445. 10.1016/j.phrs.2018.12.001 30528973

[B161] Rampino A. Marakhovskaia A. Soares-Silva T. Torretta S. Veneziani F. Beaulieu J. M. (2018). Antipsychotic Drug Responsiveness and Dopamine Receptor Signaling; Old Players and New Prospects. Front. Psychiatry 9, 702. 10.3389/fpsyt.2018.00702 30687136 PMC6338030

[B162] Rao N. Northoff G. Tagore A. Rusjan P. Kenk M. Wilson A. (2019). Impaired Prefrontal Cortical Dopamine Release in Schizophrenia During a Cognitive Task: A [11C]FLB 457 Positron Emission Tomography Study. Schizophr. Bull. 45 (3), 670–679. 10.1093/schbul/sby076 29878197 PMC6483585

[B163] Ren K. Guo B. Dai C. Yao H. Sun T. Liu X. (2017). Striatal Distribution and Cytoarchitecture of Dopamine Receptor Subtype 1 and 2: Evidence from Double-Labeling Transgenic Mice. Front. Neural Circuits 11, 57. 10.3389/fncir.2017.00057 28860974 PMC5562971

[B164] Reynolds L. M. Pokinko M. Torres-Berrio A. Cuesta S. Lambert L. C. Del Cid Pellitero E. (2018). DCC Receptors Drive Prefrontal Cortex Maturation by Determining Dopamine Axon Targeting in Adolescence. Biol. Psychiatry 83 (2), 181–192. 10.1016/j.biopsych.2017.06.009 28720317 PMC5723533

[B165] Robinson S. E. Sohal V. S. (2017). Dopamine D2 Receptors Modulate Pyramidal Neurons in Mouse Medial Prefrontal Cortex through a Stimulatory G-Protein Pathway. J. Neurosci. 37 (42), 10063–10073. 10.1523/JNEUROSCI.1893-17.2017 28912160 PMC5647767

[B166] Robinson B. G. Cai X. Wang J. Bunzow J. R. Williams J. T. Kaeser P. S. (2019). RIM is essential for stimulated but not spontaneous somatodendritic dopamine release in the midbrain. Elife 8. 10.7554/eLife.47972 PMC6754207 31486769

[B167] Robison A. J. Thakkar K. N. Diwadkar V. A. (2020). Cognition and Reward Circuits in Schizophrenia: Synergistic, Not Separate. Biol. Psychiatry 87 (3), 204–214. 10.1016/j.biopsych.2019.09.021 31733788 PMC6946864

[B168] Rodriguez-Ruiz M. Moreno E. Moreno-Delgado D. Navarro G. Mallol J. Cortes A. (2017). Heteroreceptor Complexes Formed by Dopamine D1, Histamine H3, and N-Methyl-D-Aspartate Glutamate Receptors as Targets to Prevent Neuronal Death in Alzheimer’s Disease. Mol. Neurobiol. 54 (6), 4537–4550. 10.1007/s12035-016-9995-y 27370794

[B169] Rossi M. Fasciani I. Marampon F. Maggio R. Scarselli M. (2017). The First Negative Allosteric Modulator for Dopamine D2 and D3 Receptors, SB269652 May Lead to a New Generation of Antipsychotic Drugs. Mol. Pharmacol. 91 (6), 586–594. 10.1124/mol.116.107607 28265019 PMC5438131

[B170] Saffari R. Grotefeld K. Kravchenko M. Zhang M. Zhang W. (2019). Calretinin(+)-neurons-mediated GABAergic inhibition in mouse prefrontal cortex. Prog. Neuropsychopharmacol. Biol. Psychiatry 94, 109658. 10.1016/j.pnpbp.2019.109658 31145926

[B171] Sahlholm K. Gomez-Soler M. Valle-Leon M. Lopez-Cano M. Taura J. J. Ciruela F. (2018). Antipsychotic-Like Efficacy of Dopamine D2 Receptor-Biased Ligands is Dependent on Adenosine A2A Receptor Expression. Mol. Neurobiol. 55 (6), 4952–4958. 10.1007/s12035-017-0696-y 28779351

[B172] Sala-Bayo J. Fiddian L. Nilsson S. R. O. Hervig M. E. McKenzie C. Mareschi A. (2020). Dorsal and ventral striatal dopamine D1 and D2 receptors differentially modulate distinct phases of serial visual reversal learning. Neuropsychopharmacology 45 (5), 736–744. 10.1038/s41386-020-0612-4 31940660 PMC7075980

[B173] Sallis H. M. Croft J. Havdahl A. Jones H. J. Dunn E. C. Davey Smith G. (2020). Genetic liability to schizophrenia is associated with exposure to traumatic events in childhood. Psychol. Med., 1–8. 10.1017/S0033291720000537 PMC8381289 32234096

[B174] Sekiguchi H. Pavey G. Dean B. (2019). Altered levels of dopamine transporter in the frontal pole and dorsal striatum in schizophrenia. NPJ Schizophr. 5 (1), 20. 10.1038/s41537-019-0087-7 31792225 PMC6888821

[B175] Seney M. L. Cahill K. Enwright J. Logan R. W. Huo Z. Zong W. (2019). Diurnal rhythms in gene expression in the prefrontal cortex in schizophrenia. Nat. Commun. 10 (1), 3355. 10.1038/s41467-019-11335-1 31399567 PMC6689017

[B176] Shi S. Leites C. He D. Schwartz D. Moy W. Shi J. (2014). MicroRNA-9 and microRNA-326 regulate human dopamine D2 receptor expression, and the microRNA-mediated expression regulation is altered by a genetic variant. J. Biol. Chem. 289 (19), 13434–13444. 10.1074/jbc.M113.535203 24675081 PMC4036351

[B177] Shioda N. Yabuki Y. Wang Y. Uchigashima M. Hikida T. Sasaoka T. (2017). Endocytosis following dopamine D2 receptor activation is critical for neuronal activity and dendritic spine formation via Rabex-5/PDGFRbeta signaling in striatopallidal medium spiny neurons. Mol. Psychiatry 22 (8), 1205–1222. 10.1038/mp.2016.200 27922607

[B178] Shioda N. (2017). Dopamine D2L receptor-interacting proteins regulate dopaminergic signaling. J. Pharmacol. Sci. 135, 51–54. 10.1016/j.jphs.2017.10.002 29107444

[B179] Sialana F. J. Wang A. L. Fazari B. Kristofova M. Smidak R. Trossbach S. V. (2018). Quantitative Proteomics of Synaptosomal Fractions in a Rat Overexpressing Human DISC1 Gene Indicates Profound Synaptic Dysregulation in the Dorsal Striatum. Front. Mol. Neurosci. 11, 26. 10.3389/fnmol.2018.00026 29467617 PMC5808171

[B180] Simpson E. H. Kellendonk C. (2017). Insights About Striatal Circuit Function and Schizophrenia From a Mouse Model of Dopamine D2 Receptor Upregulation. Biol. Psychiatry 81 (1), 21–30. 10.1016/j.biopsych.2016.07.004 27720388 PMC5121031

[B181] Slifstein M. Abi-Dargham A. (2018). Is it Pre- or Postsynaptic? Imaging Striatal Dopamine Excess in Schizophrenia. Biol. Psychiatry 83 (8), 635–637. 10.1016/j.biopsych.2018.02.015 29559095

[B182] Slifstein M. Abi-Dargham A. Girgis R. R. Suckow R. F. Cooper T. B. Divgi C. R. (2020). Binding of the D3-preferring antipsychotic candidate F17464 to dopamine D3 and D2 receptors: a PET study in healthy subjects with [(11)C]-(+)-PHNO. Psychopharmacol. (Berl) 237 (2), 519–527. 10.1007/s00213-019-05387-w 31773210

[B183] Soares-Cunha C. Coimbra B. David-Pereira A. Borges S. Pinto L. Costa P. (2016). Activation of D2 dopamine receptor-expressing neurons in the nucleus accumbens increases motivation. Nat. Commun. 7, 11829. 10.1038/ncomms11829 27337658 PMC4931006

[B184] Song M. R. Lee S. W. (2020). Dynamic resource allocation during reinforcement learning accounts for ramping and phasic dopamine activity. Neural Netw. 126, 95–107. 10.1016/j.neunet.2020.03.005 32203877

[B185] Sonnenschein S. F. Grace A. A. (2020). Insights on current and novel antipsychotic mechanisms from the MAM model of schizophrenia. Neuropharmacology 163, 107632. 10.1016/j.neuropharm.2019.05.009 31077730 PMC6842083

[B186] Stepien M. Manoliu A. Kubli R. Schneider K. Tobler P. N. Seifritz E. (2018). Investigating the association of ventral and dorsal striatal dysfunction during reward anticipation with negative symptoms in patients with schizophrenia and healthy individuals. PloS One 13 (6), e0198215. 10.1371/journal.pone.0198215 29912880 PMC6005482

[B187] Su P. Li S. Chen S. Lipina T. V. Wang M. Lai T. K. (2014). A dopamine D2 receptor-DISC1 protein complex may contribute to antipsychotic-like effects. Neuron 84 (6), 1302–1316. 10.1016/j.neuron.2014.11.007 25433637

[B188] Suh Y. Noh S. J. Lee S. Suh B. K. Lee S. B. Choi J. (2019). Dopamine D1 Receptor (D1R) Expression Is Controlled by a Transcriptional Repressor Complex Containing DISC1. Mol. Neurobiol. 56 (10), 6725–6735. 10.1007/s12035-019-1566-6 30915712 PMC6728282

[B189] Sultana R. Lee C. C. (2020). Expression of Behavioral Phenotypes in Genetic and Environmental Mouse Models of Schizophrenia. Front. Behav. Neurosci. 14, 29. 10.3389/fnbeh.2020.00029 32184711 PMC7058961

[B190] Sun N. Zhang X. Guo S. Le H. T. Zhang X. Kim K. M. (2018). Molecular mechanisms involved in epidermal growth factor receptor-mediated inhibition of dopamine D3 receptor signaling. Biochim. Biophys. Acta Mol. Cell Res. 1865 (9), 1187–1200. 10.1016/j.bbamcr.2018.06.001 29885323

[B191] Takeuchi S. Hida H. Uchida M. Naruse R. Yoshimi A. Kitagaki S. (2019). Blonanserin ameliorates social deficit through dopamine-D3 receptor antagonism in mice administered phencyclidine as an animal model of schizophrenia. Neurochem. Int. 128, 127–134. 10.1016/j.neuint.2019.04.008 30998952

[B192] Tan T. Wang W. Williams J. Ma K. Cao Q. Yan Z. (2019). Stress Exposure in Dopamine D4 Receptor Knockout Mice Induces Schizophrenia-Like Behaviors via Disruption of GABAergic Transmission. Schizophr. Bull. 45 (5), 1012–1023. 10.1093/schbul/sby163 30476265 PMC6737476

[B193] Tan L. Zhou Q. Yan W. Sun J. Kozikowski A. P. Zhao S. (2020). Design and Synthesis of Bitopic 2-Phenylcyclopropylmethylamine (PCPMA) Derivatives as Selective Dopamine D3 Receptor Ligands. J. Med. Chem. 63 4579–4602. 10.1021/acs.jmedchem.9b01835 32282200

[B194] Terrillion C. E. Dao D. T. Cachope R. Lobo M. K. Puche A. C. Cheer J. F. (2017). Reduced levels of Cacna1c attenuate mesolimbic dopamine system function. Genes Brain Behav. 16 (5), 495–505. 10.1111/gbb.12371 28186690 PMC5457318

[B195] Thal D. M. Glukhova A. Sexton P. M. Christopoulos A. (2018). Structural insights into G-protein-coupled receptor allostery. Nature 559 (7712), 45–53. 10.1038/s41586-018-0259-z 29973731

[B196] Tomasella E. Bechelli L. Ogando M. B. Mininni C. Di Guilmi M. N. De Fino F. (2018). Deletion of dopamine D2 receptors from parvalbumin interneurons in mouse causes schizophrenia-like phenotypes. Proc. Natl. Acad. Sci. U.S.A. 115 (13), 3476–3481. 10.1073/pnas.1719897115 29531031 PMC5879696

[B197] Torretta S. Rampino A. Basso M. Pergola G. Di Carlo P. Shin J. H. (2020). NURR1 and ERR1 Modulate the Expression of Genes of a DRD2 Coexpression Network Enriched for Schizophrenia Risk. J. Neurosci. 40 (4), 932–941. 10.1523/JNEUROSCI.0786-19.2019 31811028 PMC6975285

[B198] Tseng H. H. Chen K. C. Chen P. S. Lee I. H. Chang W. H. Yao W. J. (2017). Dopamine transporter availability in drug-naive patients with schizophrenia and later psychotic symptoms severity. Schizophr. Res. 190, 185–186. 10.1016/j.schres.2017.03.036 28364963

[B199] Tseng H. H. Watts J. J. Kiang M. Suridjan I. Wilson A. A. Houle S. (2018). Nigral Stress-Induced Dopamine Release in Clinical High Risk and Antipsychotic-Naive Schizophrenia. Schizophr. Bull. 44 (3), 542–551. 10.1093/schbul/sbx042 29036383 PMC5890468

[B200] Uchigashima M. Ohtsuka T. Kobayashi K. Watanabe M. (2016). Dopamine synapse is a neuroligin-2-mediated contact between dopaminergic presynaptic and GABAergic postsynaptic structures. Proc. Natl. Acad. Sci. U. S. A. 113 (15), 4206–4211. 10.1073/pnas.1514074113 27035941 PMC4839454

[B201] Urs N. M. Daigle T. L. Caron M. G. (2011). A dopamine D1 receptor-dependent beta-arrestin signaling complex potentially regulates morphine-induced psychomotor activation but not reward in mice. Neuropsychopharmacology 36 (3), 551–558. 10.1038/npp.2010.186 20980993 PMC3021093

[B202] Urs N. M. Gee S. M. Pack T. F. McCorvy J. D. Evron T. Snyder J. C. (2016). Distinct cortical and striatal actions of a beta-arrestin-biased dopamine D2 receptor ligand reveal unique antipsychotic-like properties. Proc. Natl. Acad. Sci. U.S.A. 113 (50), E8178–E8E86. 10.1073/pnas.1614347113 27911814 PMC5167191

[B203] Urs N. M. Peterson S. M. Caron M. G. (2017). New Concepts in Dopamine D2 Receptor Biased Signaling and Implications for Schizophrenia Therapy. Biol. Psychiatry 81 (1), 78–85. 10.1016/j.biopsych.2016.10.011 27832841 PMC5702557

[B204] Veselinovic T. Vernaleken I. Janouschek H. Cumming P. Paulzen M. Mottaghy F. M. (2018). The role of striatal dopamine D2/3 receptors in cognitive performance in drug-free patients with schizophrenia. Psychopharmacol. (Berl) 235 (8), 2221–2232. 10.1007/s00213-018-4916-6 29717334

[B205] Vosberg D. E. Leyton M. Flores C. (2020). The Netrin-1/DCC guidance system: dopamine pathway maturation and psychiatric disorders emerging in adolescence. Mol. Psychiatry 25 (2), 297–307. 10.1038/s41380-019-0561-7 31659271 PMC6974431

[B206] Vyas P. Hwang B. J. Brasic J. R. (2020). An evaluation of lumateperone tosylate for the treatment of schizophrenia. Expert Opin. Pharmacother. 21 (2), 139–145. 10.1080/14656566.2019.1695778 31790322

[B207] Walaas S. I. Hemmings H. C. Jr. Greengard P. Nairn A. C. (2011). Beyond the dopamine receptor: regulation and roles of serine/threonine protein phosphatases. Front. Neuroanat. 5, 50. 10.3389/fnana.2011.00050 21904525 PMC3162284

[B208] Walters S. H. Shu Z. Michael A. C. Levitan E. S. (2020). Regional Variation in Striatal Dopamine Spillover and Release Plasticity. ACS Chem. Neurosci. 11 (6), 888–899. 10.1021/acschemneuro.9b00577 32073248 PMC9668542

[B209] Walton E. Hibar D. P. van Erp T. G. M. Potkin S. G. Roiz-Santianez R. Crespo-Facorro B. (2018). Prefrontal cortical thinning links to negative symptoms in schizophrenia via the ENIGMA consortium. Psychol. Med. 48 (1), 82–94. 10.1017/S0033291717001283 28545597 PMC5826665

[B210] Waltz J. A. Xu Z. Brown E. C. Ruiz R. R. Frank M. J. Gold J. M. (2018). Motivational Deficits in Schizophrenia Are Associated With Reduced Differentiation Between Gain and Loss-Avoidance Feedback in the Striatum. Biol. Psychiatry Cognit. Neurosci. Neuroimaging. 3 (3), 239–247. 10.1016/j.bpsc.2017.07.008 29486865 PMC5833021

[B211] Wang Q. Mach R. H. Luedtke R. R. Reichert D. E. (2010). Subtype selectivity of dopamine receptor ligands: insights from structure and ligand-based methods. J. Chem. Inf. Model. 50 (11), 1970–1985. 10.1021/ci1002747 20936866 PMC4022043

[B212] Wang C. Niu M. Zhou Z. Zheng X. Zhang L. Tian Y. (2016). VPS35 regulates cell surface recycling and signaling of dopamine receptor D1. Neurobiol. Aging 46, 22–31. 10.1016/j.neurobiolaging.2016.05.016 27460146 PMC5018432

[B213] Wang J. R. Sun P. H. Ren Z. X. Meltzer H. Y. Zhen X. C. (2017). GSK-3beta Interacts with Dopamine D1 Receptor to Regulate Receptor Function: Implication for Prefrontal Cortical D1 Receptor Dysfunction in Schizophrenia. CNS Neurosci. Ther. 23 (2), 174–187. 10.1111/cns.12664 27996211 PMC6492711

[B214] Wang S. M. Han C. Lee S. J. Jun T. Y. Patkar A. A. Masand P. S. (2017). Investigational dopamine antagonists for the treatment of schizophrenia. Expert Opin. Invest. Drugs 26 (6), 687–698. 10.1080/13543784.2017.1323870 28443355

[B215] Wang S. Che T. Levit A. Shoichet B. K. Wacker D. Roth B. L. (2018). Structure of the D2 dopamine receptor bound to the atypical antipsychotic drug risperidone. Nature 555 (7695), 269–273. 10.1038/nature25758 29466326 PMC5843546

[B216] Wang L. Chen X. Wu Y. He K. Xu F. Xiao G. (2020). Intermittent theta burst stimulation (iTBS) adjustment effects of schizophrenia: Results from an exploratory outcome of a randomized double-blind controlled study. Schizophr. Res. 216, 550–553. 10.1016/j.schres.2019.12.008 31926810

[B217] Weidenauer A. Bauer M. Sauerzopf U. Bartova L. Nics L. Pfaff S. (2020). On the relationship of first-episode psychosis to the amphetamine-sensitized state: a dopamine D2/3 receptor agonist radioligand study. Transl. Psychiatry 10 (1), 2. 10.1038/s41398-019-0681-5 32066718 PMC7026156

[B218] Weinstein J. J. Chohan M. O. Slifstein M. Kegeles L. S. Moore H. Abi-Dargham A. (2017). Pathway-Specific Dopamine Abnormalities in Schizophrenia. Biol. Psychiatry 81 (1), 31–42. 10.1016/j.biopsych.2016.03.2104 27206569 PMC5177794

[B219] Weinstein J. J. (2019). Can D2 Receptor-Based Therapies Fix Presynaptic Dopamine? Biol. Psychiatry 85 (1), e1–e2. 10.1016/j.biopsych.2018.10.017 30527212 PMC9176410

[B220] Wengler K. He X. Abi-Dargham A. Horga G. (2020). Reproducibility assessment of neuromelanin-sensitive magnetic resonance imaging protocols for region-of-interest and voxelwise analyses. Neuroimage 208, 116457. 10.1016/j.neuroimage.2019.116457 31841683 PMC7118586

[B221] Wheeler D. S. Underhill S. M. Stolz D. B. Murdoch G. H. Thiels E. Romero G. (2015). Amphetamine activates Rho GTPase signaling to mediate dopamine transporter internalization and acute behavioral effects of amphetamine. Proc. Natl. Acad. Sci. U.S.A. 112 (51), E7138–E7147. 10.1073/pnas.1511670112 26553986 PMC4697400

[B222] Wieland S. Du D. Oswald M. J. Parlato R. Kohr G. Kelsch W. (2014). Phasic dopaminergic activity exerts fast control of cholinergic interneuron firing via sequential NMDA, D2, and D1 receptor activation. J. Neurosci. 34 (35), 11549–11559. 10.1523/JNEUROSCI.1175-14.2014 25164653 PMC6608407

[B223] Willner K. Vasan S. Abdijadid S. (2020). Atypical Antipsychotic Agents (Treasure Island (FL): StatPearls). 28846323

[B224] Wouters E. Marin A. R. Dalton J. A. R. Giraldo J. Stove C. (2019). Distinct Dopamine D(2) Receptor Antagonists Differentially Impact D(2) Receptor Oligomerization. Int. J. Mol. Sci. 20 (7), 1686. 10.3390/ijms20071686 PMC6480712 30987329

[B225] Wulff S. Nielsen M. O. Rostrup E. Svarer C. Jensen L. T. Pinborg L. (2020). The relation between dopamine D2 receptor blockade and the brain reward system: a longitudinal study of first-episode schizophrenia patients. Psychol. Med. 50 (2), 220–228. 10.1017/S0033291718004099 30642415

[B226] Xin J. Fan T. Guo P. Wang J. (2019). Identification of functional divergence sites in dopamine receptors of vertebrates. Comput. Biol. Chem. 83, 107140. 10.1016/j.compbiolchem.2019.107140 31715491

[B227] Xu H. Perez S. Cornil A. Detraux B. Prokin I. Cui Y. (2018). Dopamine-endocannabinoid interactions mediate spike-timing-dependent potentiation in the striatum. Nat. Commun. 9 (1), 4118. 10.1038/s41467-018-06409-5 30297767 PMC6175920

[B228] Xu W. Reith M. E. A. Liu-Chen L. Y. Kortagere S. (2019). Biased signaling agonist of dopamine D3 receptor induces receptor internalization independent of beta-arrestin recruitment. Pharmacol. Res. 143, 48–57. 10.1016/j.phrs.2019.03.003 30844536

[B229] Yamamoto K. Mirabeau O. Bureau C. Blin M. Michon-Coudouel S. Demarque M. (2013). Evolution of dopamine receptor genes of the D1 class in vertebrates. Mol. Biol. Evol. 30 (4), 833–843. 10.1093/molbev/mss268 23197594 PMC3603308

[B230] Yan L. Shamir A. Skirzewski M. Leiva-Salcedo E. Kwon O. B. Karavanova I. (2018). Neuregulin-2 ablation results in dopamine dysregulation and severe behavioral phenotypes relevant to psychiatric disorders. Mol. Psychiatry 23 (5), 1233–1243. 10.1038/mp.2017.22 28322273 PMC5608621

[B231] Yano H. Cai N. S. Xu M. Verma R. K. Rea W. Hoffman A. F. (2018). Gs- versus Golf-dependent functional selectivity mediated by the dopamine D1 receptor. Nat. Commun. 9 (1), 486. 10.1038/s41467-017-02606-w 29402888 PMC5799184

[B232] Yapo C. Nair A. G. Clement L. Castro L. R. Hellgren Kotaleski J. Vincent P. (2017). Detection of phasic dopamine by D1 and D2 striatal medium spiny neurons. J. Physiol. 595 (24), 7451–7475. 10.1113/JP274475 28782235 PMC5730852

[B233] Yu Q. Liu Y. Z. Zhu Y. B. Wang Y. Y. Li Q. Yin D. M. (2019). Genetic labeling reveals temporal and spatial expression pattern of D2 dopamine receptor in rat forebrain. Brain Struct. Funct. 224 (3), 1035–1049. 10.1007/s00429-018-01824-2 30604007 PMC6499762

[B234] Zhang J. Xu T. X. Hallett P. J. Watanabe M. Grant S. G. Isacson O. (2009). PSD-95 uncouples dopamine-glutamate interaction in the D1/PSD-95/NMDA receptor complex. J. Neurosci. 29 (9), 2948–2960. 10.1523/JNEUROSCI.4424-08.2009 19261890 PMC2693913

[B235] Zhang X. Wang W. Bedigian A. V. Coughlin M. L. Mitchison T. J. Eggert U. S. (2012). Dopamine receptor D3 regulates endocytic sorting by a Prazosin-sensitive interaction with the coatomer COPI. Proc. Natl. Acad. Sci. U. S. A. 109 (31), 12485–12490. 10.1073/pnas.1207821109 22802617 PMC3411939

[B236] Zhang X. Sun N. Zheng M. Kim K. M. (2016). Clathrin-mediated endocytosis is responsible for the lysosomal degradation of dopamine D3 receptor. Biochem. Biophys. Res. Commun. 476 (4), 245–251. 10.1016/j.bbrc.2016.05.104 27240955

[B237] Zhang C. Li Q. Meng L. Ren Y. (2020). Design of novel dopamine D2 and serotonin 5-HT2A receptors dual antagonists toward schizophrenia: An integrated study with QSAR, molecular docking, virtual screening and molecular dynamics simulations. J. Biomol. Struct. Dyn. 38 (3), 860–885. 10.1080/07391102.2019.1590244 30916624

[B238] Zhang X. Xiao W. Chen K. Zhao Y. Ye F. Tang X. (2020). Serum Epidermal Growth Factor is Low in Schizophrenia and Not Affected by Antipsychotics Alone or Combined With Electroconvulsive Therapy. Front. Psychiatry 11, 104. 10.3389/fpsyt.2020.00104 32194452 PMC7062789

[B239] Zhao W. Guo S. Linli Z. Yang A. C. Lin C. P. Tsai S. J. (2020). Functional, Anatomical, and Morphological Networks Highlight the Role of Basal Ganglia-Thalamus-Cortex Circuits in Schizophrenia. Schizophr. Bull. 46 (2), 422–431. 10.1093/schbul/sbz062 31206161 PMC7442374

[B240] Zheng M. Zhang X. Sun N. Min C. Zhang X. Kim K. M. (2016). RalA employs GRK2 and beta-arrestins for the filamin A-mediated regulation of trafficking and signaling of dopamine D2 and D3 receptor. Biochim. Biophys. Acta 1863 (8), 2072–2083. 10.4062/biomolther.2016.015 27188791

[B241] Zheng M. Zhang X. Min C. Choi B. G. Oh I. J. Kim K. M. (2016). Functional Regulation of Dopamine D(3) Receptor through Interaction with PICK1. Biomol. Ther. (Seoul) 24 (5), 475–481. 10.4062/biomolther.2016.015 27169823 PMC5012871

[B242] Zheng P. Hu M. Xie Y. Yu Y. Jaaro-Peled H. Huang X. F. (2019). Aripiprazole and haloperidol protect neurite lesions via reducing excessive D2R-DISC1 complex formation. Prog. Neuropsychopharmacol. Biol. Psychiatry 92, 59–69. 10.1016/j.pnpbp.2018.12.007 30597182

[B243] Zhou D. Xiao X. Li M. (2020). The schizophrenia risk isoform ZNF804A(E3E4) affects dendritic spine. Schizophr. Res. 218, 324–325. 10.1016/j.schres.2019.12.038 31956006

[B244] Zieba A. Zuk J. Bartuzi D. Matosiuk D. Poso A. Kaczor A. A. (2019). The Universal 3D QSAR Model for Dopamine D2 Receptor Antagonists. Int. J. Mol. Sci. 20 (18), 4555. 10.3390/ijms20184555 PMC6770028 31540025

[B245] Zuk J. Bartuzi D. Matosiuk D. Kaczor A. A. (2020). Preferential Coupling of Dopamine D2S and D2L Receptor Isoforms with Gi1 and Gi2 Proteins-In Silico Study. Int. J. Mol. Sci. 21 (2), 436. 10.3390/ijms21020436 PMC7013695 31936673

