# Supplementary material for: Dopamine Receptor Subtypes, Physiology and Pharmacology: New Ligands and Concepts in Schizophrenia
Source: Front Pharmacol. 2020 Jul 14;11:1003. doi: 10.3389/fphar.2020.01003 (PMC7379027; doi:10.3389/fphar.2020.01003)
Supplement: Supplementary file 1 [file DataSheet_1.pdf]

Supplementary material Table 1: DR subtypes properties and examples of SCZ genetic links impacting on DR expression or function.

	**D1**	**D5**	**D2S**	**D2L**	**D3**	**D4**
**DA Affinity (µM)**	2-50	0.25	0.063-20		0.05-0.4	0.025
**Signalling**	Gαs/ Gαolf	Gαs	Gαi/ Gαo	Gαi/ Gαo	Gαi/ Gαo	Gαi/ Gαo
**Gene polymorphisms**	5q35.2 2 exons 2 SPs	4p16.1 1 exon NCVs	11q23.2 9 exons; 2 SPs		3q13.31 10 exons SPs	11p15.5 4 exons NCVs
**SCZ linked Genes**						
**Netrin 1 / DCC (202)**	Dev. Control		Dev. Control	Dev. Control	Dev. Control	
**Pick1 (82)**			Co-localization		Co-localization	
**NOVA1 (203)**			Splicing	Splicing		
**ZFN804A(102)**			Low expression	Low expression		
**DNTPB1 BLOC1(204)**	No effect		Internaliz.	Internaliz.		
**DISC-1 KLF16 (93)**	Gene Repressor					

NCVs: non coding variants; SPs: splice variants

Supplementary material Table 2: Examples of recent chemical series with different DR pharmacological properties

Series (generic name)	Representative compound	DR pharmacology (EC50/IC50 range)	Reference
**Tetrahydro-pyrido-pyrimidinone derivatives**		D2 /D3 5-HT2A	(57)
**Aryl-piperazine (long-chain)**	Lurasidone<br>Trazodone	D1 D2 D3	(205)
**Pyrazolyl- (tetra) or dihydroisoquinolines**	DETQ (187)	D1positive allosteric modulator (nM)	(206, 207)
**Non-cathecol pyrido-furan derivatives**	PF-06649751 (208)	D1 /D5 biased agonists (pM - nM)	(209)
**Benzofurans**	O4LE7	D2 > D3 agonist (pM)	(190)
**2-Phenyl-cyclopropylmethylamine analogues**		D3 agonist, antagonists, bitopic D2 selective. 5-HT2C (nM)	(196)
**Nitro benzylderivatives**	Dichloro-eticlopride	D2/D3 antagonist (pM)	(194)
**C3-substituted (-)-stepholidine derivatives**		D3 bitopic ligands > D2 (nM)	(198)
